# Supplementary material for: Hierarchical organization of urban mobility and its connection with city livability
Source: Nat Commun. 2019 Oct 23;10:4817. doi: 10.1038/s41467-019-12809-y (PMC6811587; doi:10.1038/s41467-019-12809-y)
Supplement: Supplementary file 2 — Supplementary Information [file 41467_2019_12809_MOESM2_ESM.pdf]

Supplementary Information
Hierarchical organization of urban mobility and its connection with city livability

A. Bassolas et al.

List of Figures

1 Shape of CBSA's . . . 4
2 Set of worldwide cities . . . 6
3 Degree and Trip-length distributions . . . 8
4 Symmetry of in- and out-flows . . . 9
5 Calculation of hotspots and distributions of nodes and flow per-level . . . 10
6 Majority of flow incorporated in first three hotspot levels . . . 11
7 Flows between same-level hotspots compared to Φ. . . . 11
8 Flow-hierarchy as a function of city boundaries . . . 13
9 Relation between Φ calculated with the original S2 cells and grids of larger size in US cities. The cell sizes are **a** 2 km x 2 km, **b** 3 km x 3 km and **c** 4 km x 4 km . . . 13
10 Relation between Φ calculated with our Location History dataset and from the commuting in US cities . . . 14
11 Comparison of the spatial distribution of hotspot levels when considering all the urban mobility or only the commuting in New York City . . . 14
12 Difference between cell hotspot levels with commuting and Location History data . . . 15
13 Comparison of the flow-hierarchy Φ calculated from trip distribution models . . . 16
14 Comparison of the matrix of flows between hotspots obtained for each of the trip distribution models using the node outflow as input . . . 17
15 Comparison of Φ obtained for the nonlinear gravity model and from the commuting data 17
16 Connecting Φ to population-mixing in US cities . . . 18
17 Population-mixing in US cities as a function of hierarchical structure. Average flow entropy $\langle H \rangle$ as a function of Φ. . . . 19
18 Correlation of flow-hierarchy with urban indicators excluding New York City . . . 21
19 Relation between Φ and other transport metrics . . . 22
20 Relation between Φ and exposure rate of traffic fatal injuries. . . . 23
21 Relation between Φ and health indicators . . . 23
22 Comparison between the spatial distribution of population density in Los Angeles and New York City. . . . 24
23 Relation between Φ and population density. . . . 24
24 Correlation of population density with urban indicators . . . 25
25 Relation between Φ and the sprawl composite index . . . 25
26 Correlation of urban sprawl with urban indicators . . . 26
27 Correlation of Φ with emissions and transport at the continental level . . . 34
28 Relation between Φ and CO_2 emissions per capita. . . . 34

List of Tables

1 Set of US cities . . . 4
2 Number of nodes and links in US cities. . . . 5
3 Set of Global cities . . . 7

4 Flow-hierarchy values for the set of global cities . . . 12
5 Number of transit stops in top hotspot levels for commuting and Location History data . 15
6 Significance (p-values) for the correlations shown in Fig. 3. . . . 21
7 Correlations as function of population threshold . . . 22
8 Temporal evolution of correlations . . . 22
9 Significance (p-values) for the correlations shown in Supplementary Fig. 26. . . . 27
10 Multivariate analysis of the modal share by car . . . 28
11 Multivariate analysis of the modal share by public transportation . . . 28
12 Multivariate analysis of the modal share by walking . . . 28
13 Multivariate analysis of the Emissions of CO . . . 29
14 Multivariate analysis of the Emissions of NO_x . . . 29
15 Multivariate analysis of the Emissions of $PM10$ . . . 29
16 Multivariate analysis of the Emissions of $PM25$ . . . 29
17 Multivariate analysis of the average distance to the closest hospital . . . 29
18 Mortality age 0-5 (all variables) . . . 30
19 Mortality age 0-5 (significant variables) . . . 30
20 Mortality age 5-25 (all variables) . . . 30
21 Mortality age 5-25 (significant variables) . . . 31
22 Mortality by COPD (all variables) . . . 31
23 Mortality by COPD (significant variables) . . . 31
24 Mortality by all type of strokes (all variables) . . . 32
25 Mortality by all type of strokes (significant variables) . . . 32
26 Mortality by ischemic stroke (all variables) . . . 32
27 Mortality by ischemic stroke (significant variables) . . . 32
28 Hospitalization by ischemic stroke (significant variables) . . . 33
29 Mortality by transport fatalities (all variables) . . . 33
30 Mortality by transport fatalities (significant variables) . . . 33

Supplementary Note 1: Mobility data

Trip flows

We use aggregate mobility data from Location History. This anonymous aggregated data comes from users who opted-in to share their location data, which already is a vital source of information for estimates of live traffic and parking availability [1]. Due to the popularity of Android phones, this data set includes minute-resolution location data for 5% of the world's population. We apply machine learning to anonymized logs data to segment a raw GPS trace into semantic trips [2]. All anonymized trips are processed in aggregate to extract their origin and destination location and time. For example, if n users traveled from location a to location b within time interval t, the corresponding cell (a, b, t) in the tensor would be $n \pm e$, where e is Laplacian noise to provide strong differential privacy guarantees. This means that someone trying to determine whether a user was included in this dataset would at best improve their level of certainty over a random guess by $\sim 16\%$. Note that this models the most conservative scenario and that attempting to get precise data about a user is, in general, significantly harder. This design protects individual data from being manually inspected and only heavily aggregated flows of populations are shared. Furthermore, the dataset is generated only once and only mobility flows involving sufficiently large number of accounts are processed by the model. This design protects individual data from being manually inspected and only heavily aggregated flows of populations are shared (at the level of counties, states, large S2 cells). The underlying data is already used as a vital source of information for estimates of live traffic and parking availability. This process provides strong privacy guarantees by bounding the posterior probability—as learned by analyzing the dataset—that any particular user's data was used in producing an output. Furthermore, only flows involving a sufficient number of individuals are processed by the model ($n > 100$). No individual user data is ever inspected or modeled, only heavily aggregated flows of large populations. The data corresponds to weekly aggregation of flows for the year 2016.

S2 cells

S2 cells are a space tessellation that divide Earth in cells of similar area and was first developed by Eric Veach in 2005 at Google. The system of cells is hierarchical, with a total of thirty levels of successive smaller sizes that fit one inside the other. The earth is placed inside a cube and is then projected into each face with a non-linear transformation to preserve the areas. The cube is then tessellated in cells of varying size depending on the desired level. Finally, the Hilbert curve (a fractal-like space filling curve that maps a 2D space to 1D) is used to index each cell, being fast to encode and decode, while preserving spatial locality. Further information can be found in [3]. The mobility data corresponds to trip-flows between level-13 S2 cells, with sizes ranging between $0.76km^2$ and $1.59km^2$.

Supplementary Note 2: List of cities

In this section, we list the considered cities and the definition of their spatial boundaries. Due to easier access to urban indicators of cities and their standardized definition within the same country, we first describe US cities and then their global counterparts. The cities studied include metropolitan and micropolitan areas of the US and the most populated worldwide metropolitan areas excluding Chinese cities, where we have incomplete data. When studying urban structure and its relation with urban indicators, the definition of city boundaries can strongly influence the findings [4, 5]. Consequently the boundaries delineated for US cities are different when analyzed within the context of the US and when comparing them to worldwide cities. In the former case, metrics are calculated according to boundaries defined by the US census, while in the latter we use the ones outlined by the OECD.

US cities

The definition of metropolitan areas used for US cities correspond to Core Based Statistical Areas (CBSA), defined by the Office of Management and Budget (OMB) and used in the census [6]. The boundaries of the metropolitan and micropolitan areas are delimited by aggregating counties associated with an urban center with more than 10^4 inhabitants together with adjacent ones strongly connected to the core by commuting flows.

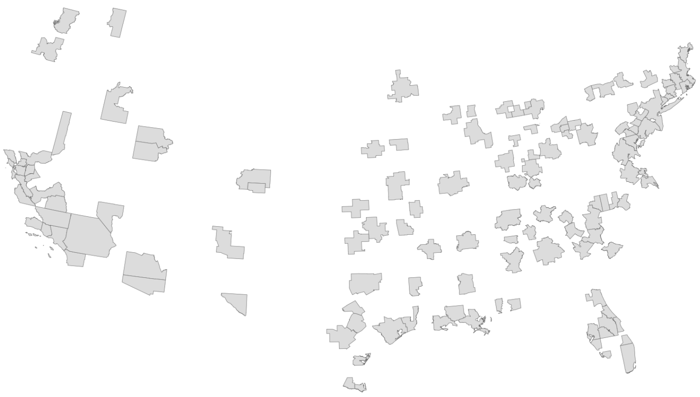

Supplementary Figure 1: Shape of Core Based Statistical Areas (CBSA) in our data

The complete list of metropolitan areas (127 cities) is detailed in Supplementary Table 1, while their shapes are shown in Supplementary Fig. 1 (obtained from [7]). Having determined the boundaries of the metropolitan areas, the mobility network of each city is constructed by including all the S2 cells inside the urban area as nodes, and trip-flows between them as weighted edges.

City	Population	City	Population	City	Population	City	Population
Akron	703	Albany	870	Albuquerque	887	Allentown	821
Asheville	424	Atlanta	5286	Augusta	564	Austin	1716
Bakersfield	839	Baltimore	2710	Baton Rouge	802	Beaumont	403
Birmingham	1128	Boise City	616	Boston	4552	Bridgeport	916
Brownsville	406	Buffalo	1135	Canton	404	Cape Coral	618
Charleston	664	Charlotte	2217	Chattanooga	528	Chicago	9461
Cincinnati	2114	Cleveland	2077	Colorado Springs	645	Columbia	767
Columbus	1901	Corpus Christi	428	Dallas	6426	Dayton	799
Deltona	590	Denver	2543	Des Moines	569	Detroit	4296
Durham	504	El Paso	804	Fayetteville	463	Flint	425
Fort Wayne	416	Fresno	930	Grand Rapids	988	Greensboro	723
Greenville	824	Harrisburg	549	Hartford	1212	Houston	5920
Huntsville	417	Indianapolis	1887	Jackson	567	Jacksonville	1345
Kansas City	2009	Killeen	405	Knoxville	837	Lafayette	466
Lakeland	602	Lancaster	519	Lansing	464	Las Vegas	1951
Lexington	472	Little Rock	699	Los Angeles	12828	Louisville	1235
Madison	605	Manchester	400	McAllen	774	Memphis	1324
Miami	5564	Milwaukee	1555	Minneapolis	3348	Mobile	412
Modesto	514	Nashville	1670	New Haven	862	New Orleans	1189
New York	19567	North Port	702	Ogden	597	Oklahoma City	1252
Omaha	865	Orlando	2134	Oxnard	823	Palm Bay	543
Pensacola	448	Philadelphia	5965	Phoenix	4192	Pittsburgh	2356
Port St. Lucie	424	Portland (OR)	2226	Portland (ME)	514	Providence	1600
Raleigh	1130	Reading	411	Reno	425	Richmond	1208
Riverside	4224	Rochester	1079	Sacramento	2149	Salinas	415
Salt Lake City	1087	San Antonio	2142	San Diego	3095	San Francisco	4335
San Jose	1836	Santa Maria	423	Santa Rosa	483	Scranton	563
Seattle	3439	Shreveport	439	Spokane	527	Springfield (MO)	436
Springfield (MA)	621	St. Louis	2787	Stockton	685	Syracuse	662
Tampa	2783	Toledo	610	Tucson	980	Tulsa	937
Vallejo	413	Virginia Beach	1676	Visalia	442	Washington	5636
Wichita	630	Winston	640	Worcester	916	York	434
Youngstown	565						

Supplementary Table 1: Set of US cities and their population in thousands of inhabitants.

We provide in Supplementary Table 2 the number of nodes and links in US cities.

City	Cells	Links	City	Cells	Links	City	Cells	Links	City	Cells	Links
New Haven	394	4479	Lexington	322	3572	Youngstown	296	2270	Syracuse	288	2894
Baton Rouge	472	4646	Colorado Springs	470	6743	Bakersfield	334	5592	Visalia	201	2430
Akron	506	3969	Albany	439	3932	Albuquerque	519	8938	Allentown	406	4232
Asheville	253	2291	Atlanta	4156	46333	Augusta	306	3175	Austin	1110	17247
Bakersfield	334	5592	Baltimore	1433	19581	Baton Rouge	472	4646	Beaumont	320	3336
Birmingham	636	5696	Boise City	430	5427	Boston	1945	23220	Bridgeport	356	4428
Brownsville	270	4437	Buffalo	595	7178	Canton	234	2231	Cape Coral	588	5913
Charleston	577	6385	Charlotte	1573	16250	Chattanooga	334	2840	Chicago	5557	71939
Cincinnati	1441	13188	Cleveland	1271	13489	Colorado Springs	470	6743	Columbia	506	5067
Columbus	1099	12097	Corpus Christi	335	4919	Dallas	4245	65381	Dayton	642	5916
Deltona	332	3805	Denver	1582	26083	Des Moines	500	4981	Detroit	2880	29038
Durham	276	3248	El Paso	503	9718	Fayetteville	287	2980	Flint	309	2160
Fort Wayne	278	2663	Fresno	465	7284	Grand Rapids	460	4455	Greensboro	366	4320
Greenville	513	4615	Harrisburg	351	3285	Hartford	614	5740	Houston	4014	59051
Huntsville	265	2655	Indianapolis	1343	13168	Jackson	276	2079	Jacksonville	1045	12063
Kansas City	1597	16938	Killeen	271	3723	Knoxville	516	4268	Lafayette	196	1745
Lakeland	513	4779	Lancaster	302	2437	Lansing	268	2669	Las Vegas	946	21535
Lexington	322	3572	Little Rock	419	4310	Los Angeles	3959	107810	Louisville	826	9364
Madison	318	3213	Manchester	186	1832	McAllen	635	8673	Memphis	912	9290
Miami	2275	55179	Milwaukee	948	10786	Minneapolis	2354	21556	Mobile	283	2565
Modesto	264	4138	Nashville	1128	11928	New Haven	394	4479	New Orleans	644	10297
New York	6213	110798	North Port	499	5868	Ogden	453	5012	Oklahoma City	803	9906
Omaha	585	6499	Orlando	1751	26738	Oxnard	377	5837	Palm Bay	462	4876
Pensacola	358	3334	Philadelphia	2868	36644	Phoenix	2383	38830	Pittsburgh	1098	9807
Port St. Lucie	353	3555	Portland	1209	18955	Portland1	164	1339	Providence	816	9321
Raleigh	847	8967	Reading	142	1754	Reno	275	3852	Richmond	812	8639
Riverside	2687	38968	Rochester	496	5370	Sacramento	1279	17531	Salinas	240	3053
Salisbury	317	2486	Salt Lake City	642	10191	San Antonio	1286	22012	San Diego	1464	26469
San Francisco	1819	33974	San Jose	735	15799	Santa Maria	145	1685	Santa Rosa	242	3203
Scranton	272	2574	Seattle	2071	30966	Shreveport	228	2323	Spokane	290	3951
Springfield	242	2698	Springfield1	313	3517	St. Louis	1829	17665	Stockton	376	5577
Syracuse	288	2894	Tampa	1908	27540	Toledo	416	4073	Tucson	560	8392
Tulsa	561	6133	Vallejo	244	3318	Virginia Beach	1079	15051	Visalia	201	2430
Washington	2830	43166	Wichita	413	4619	Winston	304	2928	Worcester	269	2566
York	151	1588	Youngstown	296	2270						

Supplementary Table 2: Number of nodes and links in US cities.

Worldwide cities

The Organization for Economic Cooperation and Development (OECD) provides boundaries for functional urban areas in member countries [8]. Using a gridded population dataset, urban cores are defined as clusters of adjoining grid cells with a population density above a certain threshold—1500 inhabitants per km^2 for all regions except Mexico and United States, where due to lower density the threshold is 1000 inhabitants per km^2. Many Asian and African cities are not part of the dataset. For these, we use data from the Atlas of Urban Expansion [9] (AOUE), which provides a definition of city boundaries based on the extension of the built-up area. Finally, for cities not included in either the OECD or AOUE lists, we define a boundary by clustering S2 cells within $4km$ of each other, up to $50km$ from the city center, until we a connected component emerges in the network. For sake of completeness all analysis conducted in this work is done for each definition of city boundary for sake of consistency. The complete list of global cities cities (172) is shown in Supplementary Table 3, together with the boundary definition for each city; Supplementary Fig. 2 shows the location of the cities on a map colored by continent.

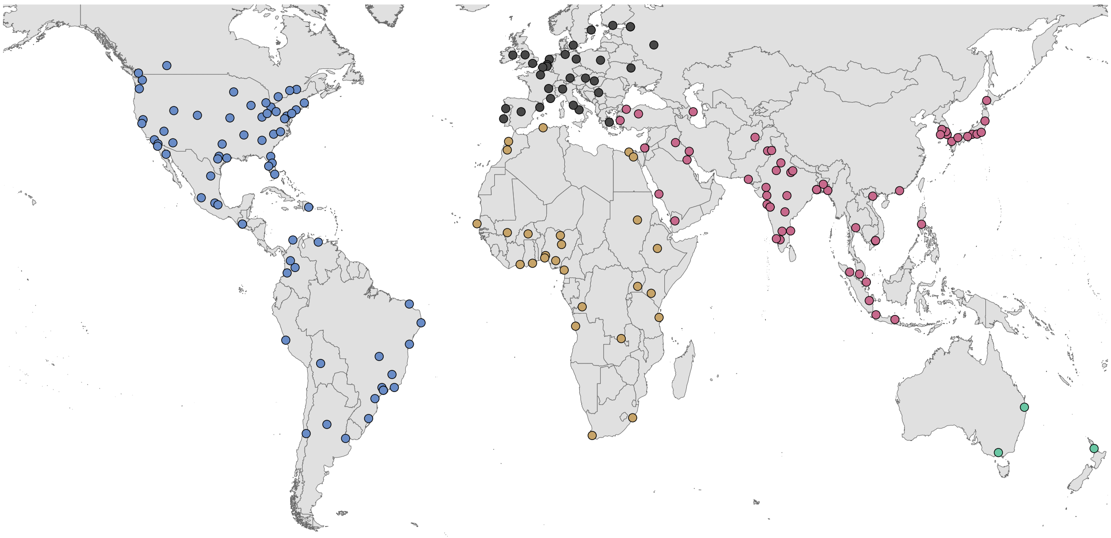

Supplementary Figure 2: Worldwide cities selected for the study, colored by continent.

City	Boundaries	City	Boundaries	City	Boundaries	City	Boundaries
Abidjan	Other	Abuja	Other	Accra	AOUE	Ahmedabad	AOUE
Alexandria	AOUE	Amsterdam	OECD	Ankara	Other	Athens	OECD
Atlanta	OECD	Auckland	AOUE	Austin	OECD	Baghdad	Other
Baltimore	OECD	Bamako	AOUE	Bandung	Other	Bangalore	Other
Bangkok	AOUE	Barcelona	OECD	Barranquilla	OECD	Belgrade	AOUE
Belo Horizonte	AOUE	Berlin	OECD	Bogota D.C.	OECD	Boston	OECD
Brasilia	Other	Brisbane	OECD	Brussels	OECD	Budapest	AOUE
Buenos Aires	AOUE	Busan	OECD	Cairo	AOUE	Calgary	OECD
Cali	OECD	Campinas	Other	Cape Town	Other	Caracas	AOUE
Charlotte	OECD	Chicago	OECD	Chittagong	Other	Cincinnati	OECD
Cleveland	OECD	Coimbatore	AOUE	Columbus	OECD	Copenhagen	OECD
Daegu	OECD	Daejeon	OECD	Dakar	Other	Dallas	OECD
Damascus	Other	Dar es Salaam	Other	Delhi	Other	Denver	OECD
Detroit	OECD	Dhaka	AOUE	Douala	Other	Dublin	OECD
Durban	Other	Faisalabad	Other	Fort Worth	OECD	Fortaleza	Other
Fukuoka	OECD	Guadalajara	OECD	Guatemala City	AOUE	Gwangju	OECD
Hanoi	Other	Hamburg	OECD	Helsinki	OECD	Hiroshima	OECD
Ho Chi Minh City	AOUE	Hong Kong	Other	Houston	OECD	Hyderabad	AOUE
Ibadan	AOUE	Istanbul	AOUE	Izmir	Other	Jaipur	AOUE
Jakarta	Other	Jiddah	Other	Johannesburg	AOUE	Kabul	AOUE
Kampala	AOUE	Kano	Other	Kanpur	AOUE	Kansas City	OECD
Karachi	AOUE	Kiev	Other	Kigali	AOUE	Kolkata	AOUE
Kuala Lumpur	Other	Kumasi	Other	Kuwait City	Other	Lagos	AOUE
Lahore	AOUE	Las Vegas	OECD	Lille	OECD	Lima	Other
Lisbon	OECD	London	OECD	Los Angeles	OECD	Luanda	AOUE
Lucknow	Other	Lyon	OECD	Madras	Other	Madrid	OECD
Manchester	OECD	Manila	AOUE	Marseille	OECD	Medan	AOUE
Medellin	OECD	Melbourne	OECD	Memphis	OECD	Mexicali	OECD
Mexico City	OECD	Miami	OECD	Milan	OECD	Minneapolis	OECD
Monterrey	OECD	Montreal	OECD	Moscow	AOUE	Mumbai	AOUE
Munich	OECD	Nagoya	OECD	Nagpur	Other	Nairobi	Other
Naples	OECD	New York	OECD	Orlando	OECD	Osaka	OECD
Ottawa-Gatineau	OECD	Ouagadougou	Other	Palembang	AOUE	Paris	OECD
Philadelphia	OECD	Phoenix	OECD	Pittsburgh	OECD	Portland	OECD
Porto	OECD	Porto Alegre	Other	Puebla	OECD	Pune	AOUE
Raleigh	OECD	Recife	Other	Rio de Janeiro	Other	Rome	OECD
Sacramento	OECD	Saint Petersburg	AOUE	Salt Lake City	OECD	Salvador	Other
San Antonio	OECD	San Diego	OECD	San Francisco	OECD	Sana'a'	Other
Santiago	OECD	Santo Domingo	Other	SaoPaulo	AOUE	Sapporo	OECD
Seattle	OECD	Sendai	OECD	Seoul	OECD	Shizuoka	Other
Singapore	AOUE	Stockholm	OECD	Surabaya	Other	Surat	Other
Sydney	OECD	Tampa	OECD	Tel Aviv	Other	Tijuana	OECD
Tokyo	OECD	Toronto	OECD	Vancouver	OECD	Vienna	OECD
Warsaw	OECD	Washington	OECD				

Supplementary Table 3: Set of global cities and the corresponding method used to calculate its boundaries.

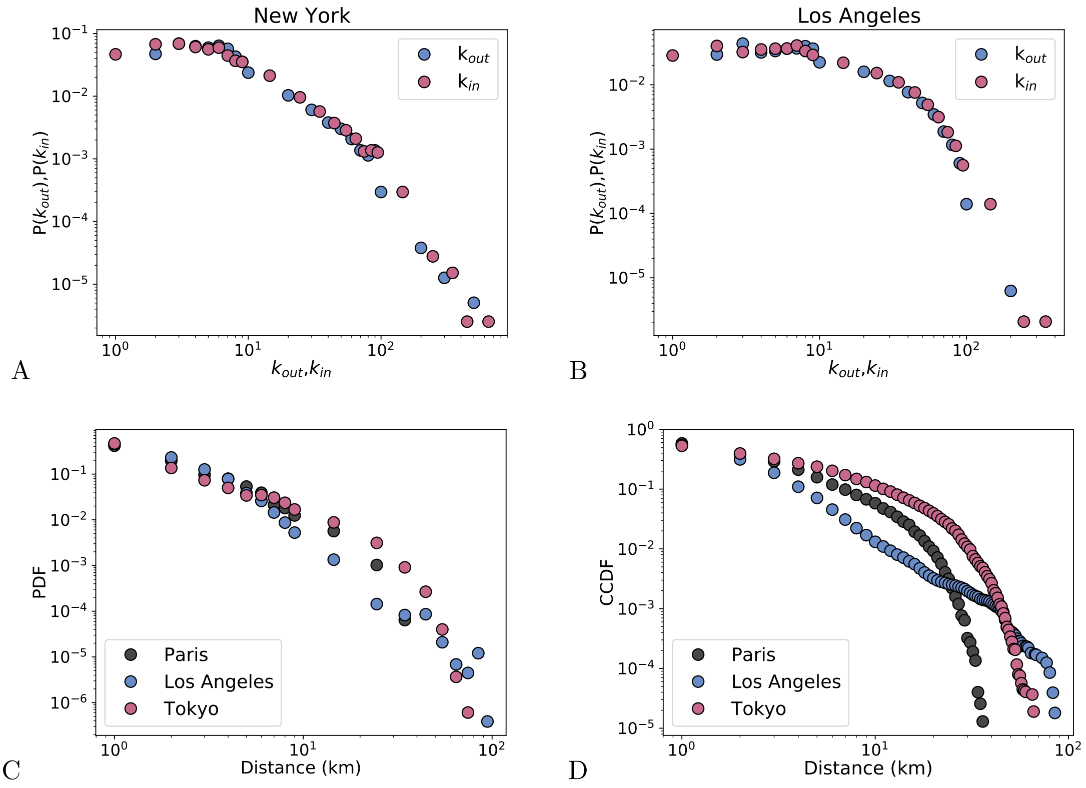

Supplementary Figure 3: In- and out-degree distributions for **a** New York and **b** Los Angeles. Distribution of trip-lengths for Paris, Los Angeles and Tokyo (pdf **c** and cdf **d**).

Supplementary Note 3: Calculation of hotspots

Network metrics

The networks consist of S2 cells as nodes, and annual trip-flows as links within the boundaries of the corresponding metropolitan areas. The links are directed and weighted, and normalized by the total number of trips in the metropolitan area for the year 2016. Supplementary Fig. 3 shows the in- and out-degree distributions for the New York and Los Angeles indicating that they are quite similar. One of the more commonly studied measures in the context of individual and urban mobility networks is the distribution of displacements [10, 11, 12]. While traditionally this is done on disaggregated data generated from Call Data Records (CDR's), the data available to us consists of population flows. Nevertheless one can make a correspondence by plotting the trip-length distribution shown in Supplementary Fig. 3, where the pdf's (a) and cdf's (b) are shown for three cities: Tokyo, Paris and Los Angeles. The observed distributions are similar to that measured in previous analysis done on disaggregated data [13, 14], with a heterogeneous power-law-like decay between $1km$ and $10km$ and a city-size dependent cut-off.

Definition

Hotspots can be broadly defined as spatial units with activity markedly higher than other in the city, defined by a threshold. This activity can be quantified through a variety of metrics including the number of individuals [15] or the population density [16]. Here, as we are concerned with mobility networks, we define the activity as the total trip-flow originating or arriving at a given node. Given the directional nature, we have two metrics: the inflow and outflow, calculated for each node i thus,

$$f_i^{out} = \sum_{j=1}^{N} w_{ij}; \qquad f_i^{in} = \sum_{j=1}^{N} w_{ji}, \tag{1}$$

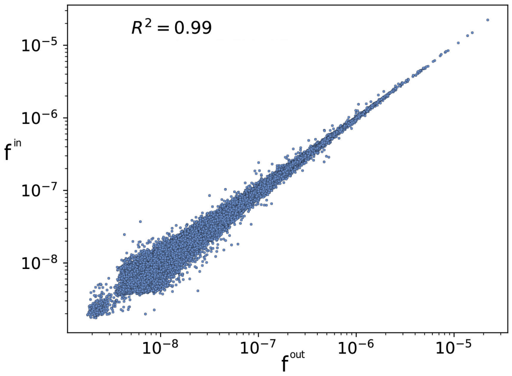

Supplementary Figure 4: Scatter plot of trip outflow and inflow aggregated over S2 cells for the 100 most populated cities in the United States.

where w_{ij} is the number of annual trips from node i to node j, normalized by the total number of annual trips in the city. Given the relatively stable population at each location, modulo noise introduced by the detection limits of the mobile technology, the in- and out-going trips are symmetric as shown in Supplementary Fig. 4, a scatter plot of f^{out} and f^{in} for all nodes in the 100 largest cities (by population) in the United States.

To determine the threshold for a cell to be labeled a hotspot we use the so-called Loubar method proposed in [15] based on the Lorenz curve for the trip outflow. The Lorenz curve is the sorted cumulative distribution of outflows and is obtained by plotting, in ascending order, the normalized cumulative number of nodes vs. the fraction of total outflow. The area between diagonal and the curve corresponds to the standard Gini coefficient widely used to measure inequality. The threshold is obtained by taking the derivative of the Lorenz curve at (1,1) and extrapolating it to the the point at which it intersects the x-axis. In our analysis, we extend the method to iteratively obtain consecutive hotspot levels. Once hotspot nodes at a level l have been extracted, they are excluded from the distribution, and the threshold is recalculated such that a new hotspot at level $l+1$ is assigned.

The procedure is illustrated for up to $l = 5$ for New York and Los Angeles in Supplementary Fig. 5a,b. The Lorenz curves are depicted in progressively transparent shades of blue as one goes down in levels, and the corresponding derivatives at $(1,1)$ from red to yellow. The shape of the outflow distribution changes the assignation of hotspot levels (Supplementary Fig. 5c,d). New York, with a more peaked distribution, has a lower number of nodes in the first hotspot levels compared to Los Angeles. The hotspot level with the maximum number of nodes assigned is shifted, being level three in Los Angeles and five in New York. In general, the majority of cities have a similar shape, with less number of nodes in high and low levels, but a high variability in the location of the peak. The total number of hotspot levels varies between cities and it is correlated with the city size or the number of nodes in the network. Despite the difference in the number distribution of nodes in hotspots levels, the distribution of the total outflow of the nodes assigned to each hotspot level is relatively homogeneous across cities. (Supplementary Fig. 5e,f). Indeed, the majority of flows are contained in the first three or four levels.

Supplementary Note 4: Flow-hierarchy Φ

Having extracted the hotspots, we next seek to quantify their interaction. Given that hotspots correspond to levels of high activity, a natural question to ask is whether the majority of the urban population are restricted to higher-level hotspots or do they transition between hotspots of varying levels of activity? We define a matrix $\mathbf{T}$ whose elements T_{ij} correspond to trips between hotspots i and j, normalized by the total number of trips in the city. Supplementary Fig. 5e,f shows that the majority of the flow is contained in the first three or four levels of hotspots, indicating that cities tend to be top-heavy with the population traveling between areas of high activity.

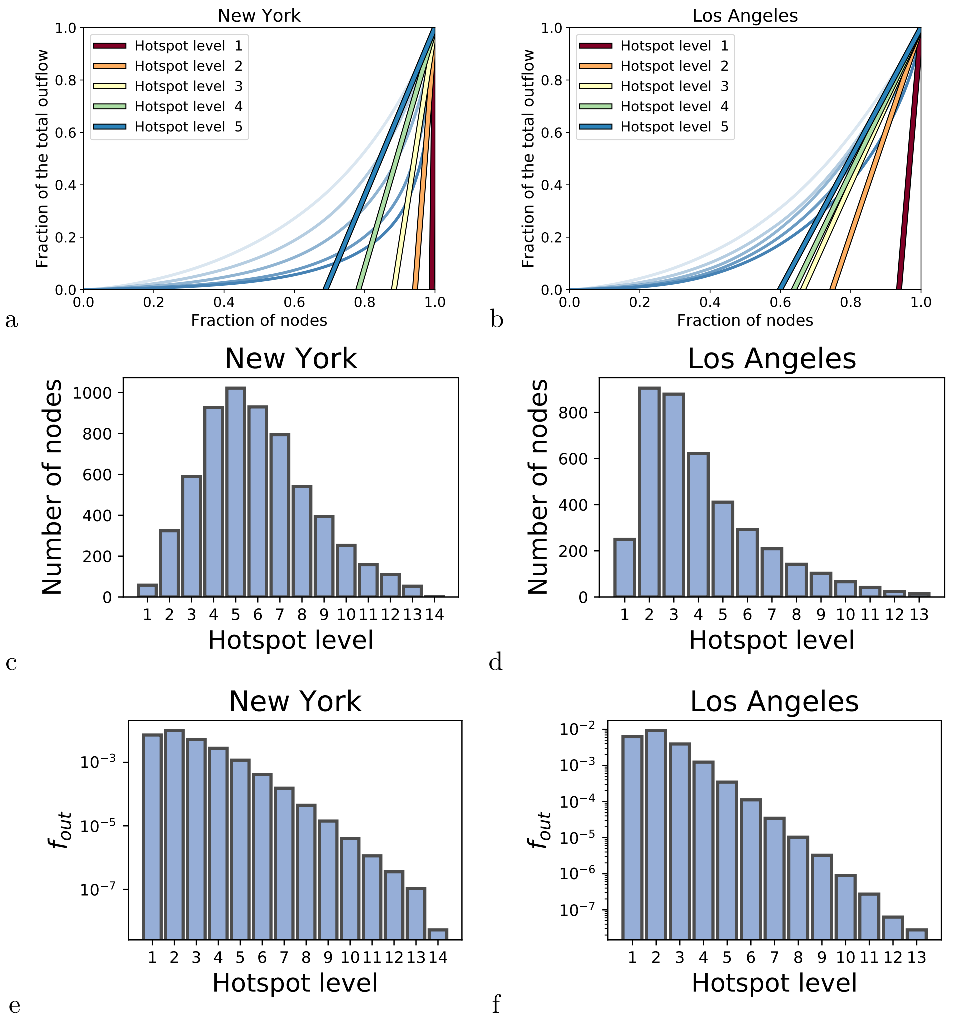

Supplementary Figure 5: Hotspot levels calculated using the Loubar method for **a** New York and **b** Los Angeles. Distribution for number of nodes per level in **c** New York and **d** Los Angeles. Distribution of outflow per level in **e** New York and **f** Los Angeles.

Definition

As a first pass, an effective way to check for this is to calculate $Tr\ \mathbf{T}$ which measures the extent to which populations traverse only within hotspots of the same level. Indeed, if all flows were contained within same-level hotspots, the matrix will be diagonal with zero entries in off-diagonal elements, and would constitute an extreme level of hierarchy. Fig. 2a,b in the main manuscript suggests that this is not the case and a significant amount of interaction occurs between hotspots of different levels. On the other hand, as Supplementary Fig. 6 indicates, the majority of interactions among hotspots of the same level, occurs at the top, as $Tr\ \mathbf{T}_{3x3}$ captures essentially all such flows.

While all cities in our dataset, seem to be top-heavy in terms of flows, they may have different levels of hierarchy depending upon the extent of interaction among the hotspot levels. Since the archetypal hierarchical structure is a tree, the extent to which cities are tree-like or flat in terms of population flow is captured by the flow-hierarchy defined as,

$$\begin{aligned} \Phi &= \textstyle\sum_{i=1}^{L-1}(T_{ii} + T_{i(i+1)} + T_{(i+1)i}) + T_{LL} \\ &= \textstyle\sum_{i,j=1}^{L} T_{ij}(\delta_{ij} + \delta_{i(j-1)} + \delta_{(i-1)j}), \end{aligned} \qquad (2)$$

corresponding to the tri-diagonal sum of the matrix $\mathbf{T}$. Here L corresponds to the total number of hotspot levels (which varies from city to city). The metric is one if the flow interaction occurs only between same-

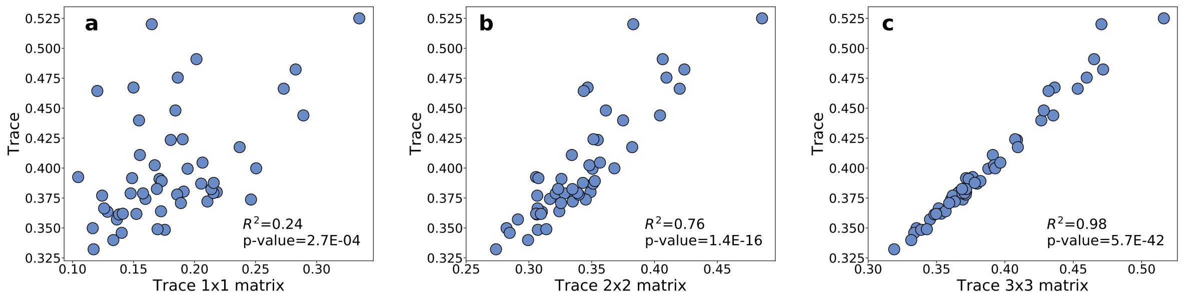

Supplementary Figure 6: Trace of the complete matrix compared to normalized flow between **a** level 1 hotspots, **b** level 1-2 hotspots, and **c** level 1-3 hotspots

or adjacent-level hotspots and close to zero if flows are flat, i.e distributed uniformly across all L hotspots. Thus Φ measures to what extent population flows in cities are tree-like. In Supplementary Fig. 7, we plot $Tr\ \mathbf{T}$ against Φ indicating a linear dependence (as expected), however, while the former captures only half the flows, Φ captures practically twice the amount of total flows in the system.

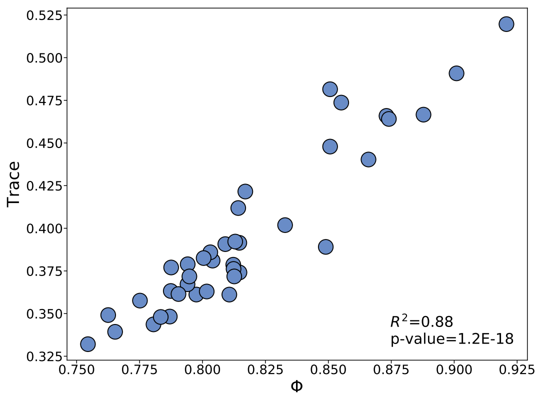

Supplementary Figure 7: Flows between same-level hotspots compared to Φ.

City	Φ	City	Φ	City	Φ	City	Φ
Ho Chi Minh City	0.954	Sana'a'	0.952	Abuja	0.951	Lahore	0.948
Luanda	0.948	Jiddah	0.948	Manila	0.948	Hanoi	0.947
Faisalabad	0.945	Kolkata	0.941	Ouagadougou	0.938	Alexandria	0.938
Lagos	0.937	Accra	0.937	Kanpur	0.936	Madras	0.936
Karachi	0.935	Mexicali	0.935	Jakarta	0.934	Kano	0.932
Porto	0.932	Lyon	0.931	Dhaka	0.931	Cairo	0.931
Surabaya	0.93	Dakar	0.93	Istanbul	0.93	Paris	0.929
Salvador	0.928	Mexico City	0.928	Coimbatore	0.927	Bangalore	0.925
Ahmedabad	0.925	Lisbon	0.922	Fortaleza	0.922	Milan	0.922
Delhi	0.921	Abidjan	0.921	Santo Domingo	0.92	Medan	0.92
Barcelona	0.919	Chittagong	0.919	Hyderabad	0.918	Guadalajara	0.918
Rome	0.918	New York	0.916	Bangkok	0.914	Jaipur	0.914
Bogota D.C.	0.913	Brussels	0.912	Lima	0.912	Surat	0.911
Barranquilla	0.91	Recife	0.91	Lucknow	0.909	Fukuoka	0.909
Marseille	0.908	Athens	0.908	Budapest	0.907	SaoPaulo	0.907
Lille	0.907	Kumasi	0.906	Bandung	0.905	Mumbai	0.905
Belo Horizonte	0.905	Dar es Salaam	0.904	Douala	0.904	Munich	0.903
Cali	0.903	Berlin	0.902	Sapporo	0.901	Kabul	0.9
Nagpur	0.9	Vienna	0.9	Philadelphia	0.9	Saint Petersburg	0.899
Tijuana	0.899	Nagoya	0.897	Moscow	0.896	Warsaw	0.894
Shizuoka	0.894	Tel Aviv	0.893	Belgrade	0.893	Hamburg	0.893
Ibadan	0.893	Puebla	0.893	Naples	0.892	Ankara	0.892
Boston	0.892	London	0.891	Copenhagen	0.891	Baghdad	0.891
Montreal	0.889	Amsterdam	0.889	Kampala	0.888	Buenos Aires	0.887
Kuala Lumpur	0.887	Monterrey	0.886	Daejeon	0.884	Porto Alegre	0.884
Palembang	0.883	Seoul	0.882	Pune	0.881	Johannesburg	0.881
Kuwait City	0.881	Hiroshima	0.88	Madrid	0.879	Chicago	0.878
Guatemala City	0.876	Izmir	0.876	Gwangju	0.875	Daegu	0.875
Osaka	0.874	Medellin	0.869	Rio de Janeiro	0.868	Toronto	0.868
Brasilia	0.866	Kiev	0.866	Manchester	0.865	San Francisco	0.864
Sendai	0.864	Caracas	0.864	Singapore	0.863	Santiago	0.863
Nairobi	0.862	Dublin	0.859	Busan	0.858	Tokyo	0.858
Kigali	0.857	Bamako	0.856	Los Angeles	0.855	Durban	0.854
Las Vegas	0.852	Campinas	0.849	Damascus	0.847	Cape Town	0.844
Melbourne	0.843	Stockholm	0.842	Ottawa-Gatineau	0.84	Calgary	0.838
Sydney	0.836	Cleveland	0.831	Miami	0.83	Baltimore	0.829
Pittsburgh	0.827	Memphis	0.826	Brisbane	0.826	Tampa	0.817
Minneapolis	0.816	Phoenix	0.816	Vancouver	0.815	Detroit	0.814
Atlanta	0.814	Dallas	0.814	Washington	0.813	Houston	0.812
San Diego	0.812	Austin	0.811	Salt Lake City	0.81	Orlando	0.807
Columbus	0.806	Sacramento	0.806	Seattle	0.803	Helsinki	0.803
Hong Kong	0.803	Fort Worth	0.799	Portland	0.798	Auckland	0.798
San Antonio	0.794	Kansas City	0.793	Denver	0.791	Raleigh	0.789
Cincinnati	0.786	Charlotte	0.771				

Supplementary Table 4: Values of Φ for the set of global cities studied.

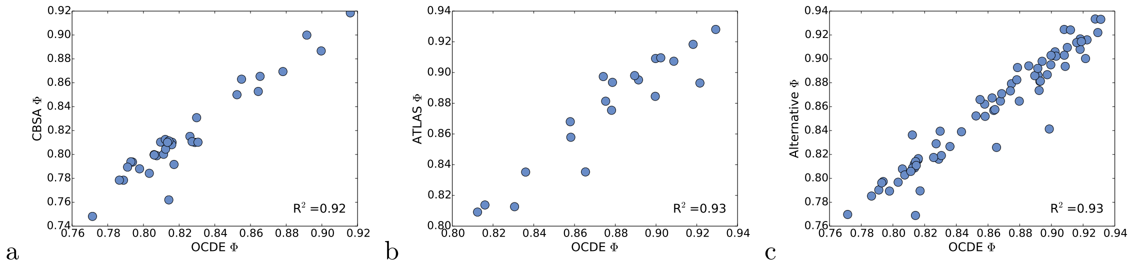

Supplementary Figure 8: Flow-hierarchy of cities using the OECD definition of functional urban areas compared to **a** US Core Based Statistical Areas, **b** Atlas of Urban Expansion and **c** the alternative definition which includes all spatially adjacent cells.

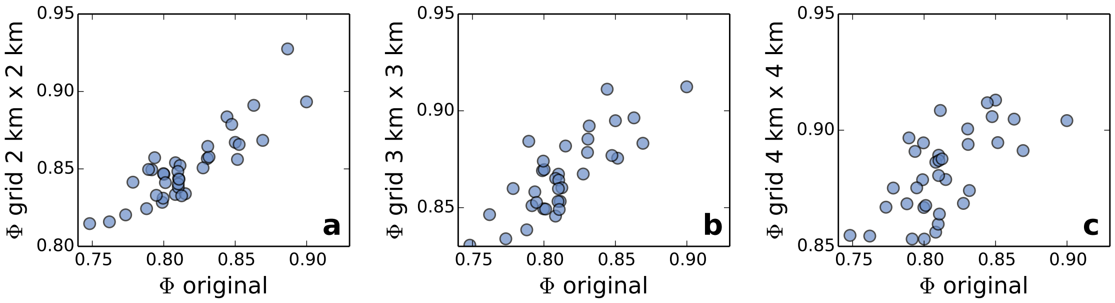

Supplementary Figure 9: Relation between Φ calculated with the original S2 cells and grids of larger size in US cities. The cell sizes are **a** 2 km x 2 km, **b** 3 km x 3 km and **c** 4 km x 4 km.

Dependence on boundary effects

It has been shown that various city metrics and its connection to urban indicators are strongly affected by the definition of city boundaries [4, 17, 18]. In order to check for the robustness of Φ we next compute the flows for different definitions of the boundary as discussed in Sec. Supplementary Note 1:.

Supplementary Fig. 8 compares the values of Φ computed from the OECD definition of boundaries against three alternative definitions: CBSA's in US cities, the ATLAS definition, and the definition based on the continuity of the cells. In all three cases, there is a strong level of agreement between the different variants of Φ . This indicates that the characterization of cities boundaries relatively stable and the metric is robust to different definitions of what constitutes a city. Note that for all figures presented in the manuscript we employ the OECD definition of boundaries.

Additionally, as a further verification, we map the Location History data into grids of progressively lower resolution in Supplementary Fig. 9 to check the stability of Φ across multiple spatial scales. As can be seen, despite the (slow) increase in value, the relative ranking of the cities is maintained indicating a robust dependence.

Supplementary Note 5: Φ from commuting data

The methodology developed through this work to quantify the hierarchical organization of mobility flows is general and can be applied to any dataset. In Supplementary Fig. 10, we show the comparison between Φ obtained from the Location History dataset and its counterpart measured from commuting data. This data at block level was obtained from the US census (`https://lehd.ces.census.gov/data`). Despite a light correlation, the differences between Φ measured from both dataset are large, which implies that the commuting mobility displays a different organization. Moreover, the mobility of commuters seems to be, in general, less hierarchical than the overall mobility of citizens.

To obtain further insights, we investigate the spatial distribution of hotspot levels in New York City using the Location History dataset and commuting (Supplementary Fig. 11). A first observation is that

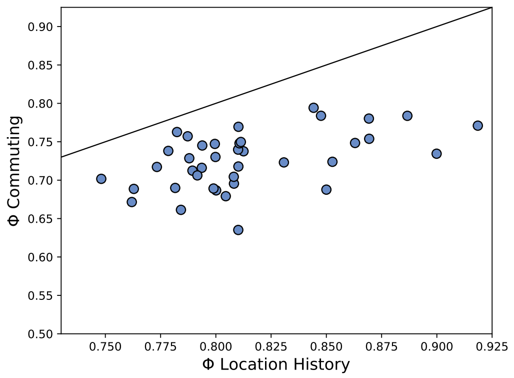

Supplementary Figure 10: Relation between Φ calculated with our Location History dataset and from the commuting in US cities

the top level hotspots are more concentrated in the case of the complete mobility. Although, in both cases the spatial distribution is relatively similar.

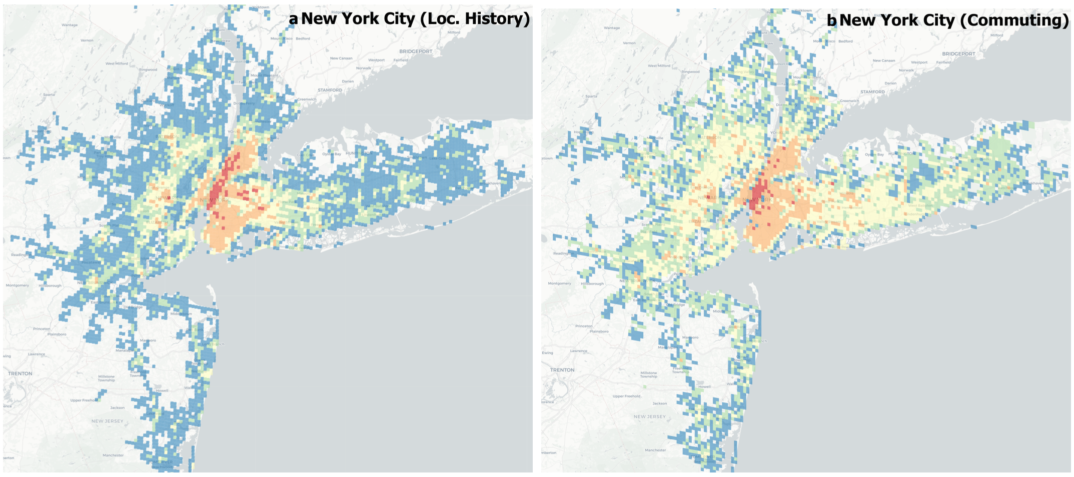

Supplementary Figure 11: Comparison of the spatial distribution of hotspot levels when considering all the urban mobility or only the commuting in New York City. Hotspot levels in New York City for **a** all the mobility and **b** only the commuting. The colors corresponding to the hotspot levels are: red (top ranking hotspots), orange (level 2), yellow (level 3), green (level 4) and blue (levels 5 or more). The underground map layout is produced using Carto. Map tiles by Carto, under CC BY 3.0. Data by OpenStreetMap, under ODbL.

To provide a more detailed comparison of both maps, we plot in Supplementary Fig. 12 the difference between hotspot levels (Commuting - Location History). Red and orange cells correspond to places that are of high level according to the Location History data and low respect to commuting. In the opposite side of the spectrum, blue and dark blue are locations with high rank in commuting and low in Location History. Yellow corresponds to cells with equivalent hotspot levels in both datasets. Blue and dark blue cells are mainly located in the suburbs, which usually correspond to residential areas. Conversely, orange and red cells can be identified as transportation hubs, parks or leisure centers.

These cells are top ranking hotspots in the overall mobility because they attract multitudes for activities such as concerts, sport activities, walking, etc, that do not require a large amount of workers in terms of servicing providing and they occur in locations as, for instance, parks, stadiums, where there are no dense residence concentrations. Examples of this type of hotspots can be seen in Supplementary Fig. 12b with the lake of Central park, the Mets and Brooklyn Nets stadiums. Other locations where the overall mobility dominates over commuting are large transportation hubs. Examples include Newark

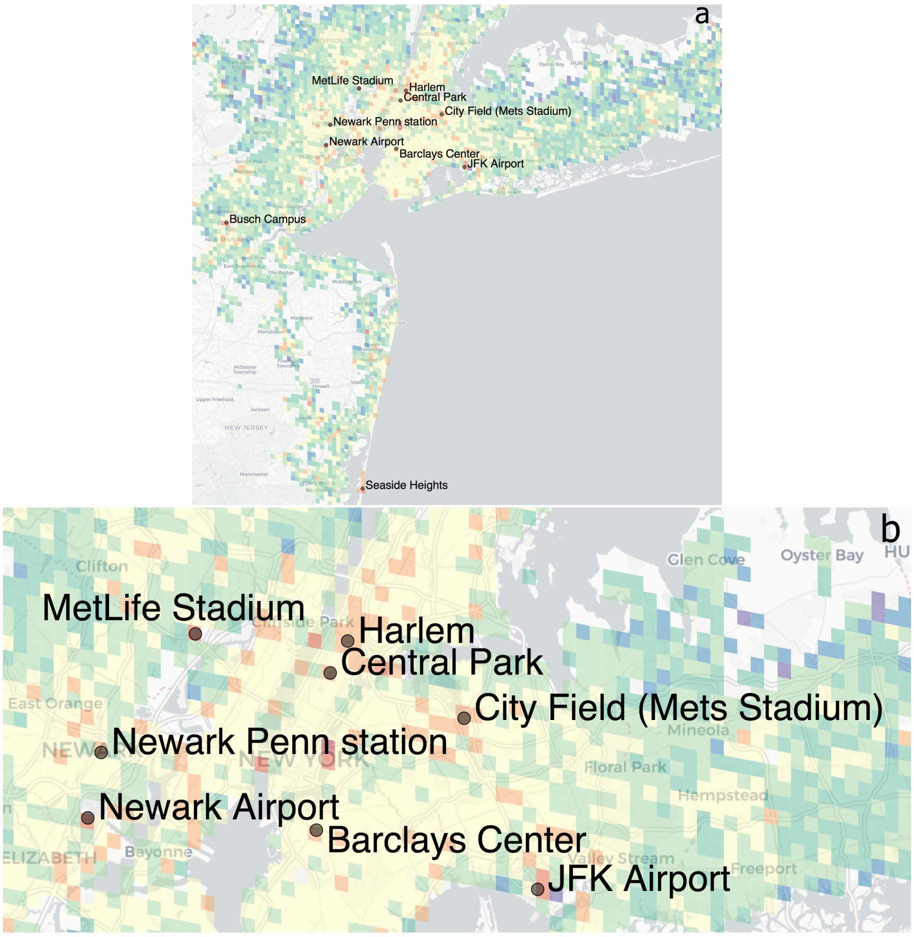

Supplementary Figure 12: Difference between cell hotspot levels with commuting and Location History data (a) for all New York City and (b) in a zoom in the main city districts. The underground map layout is produced using Carto. Map tiles by Carto, under CC BY 3.0. Data by OpenStreetMap, under ODbL.

and JFK airports and the Newark Penn Station. Other places for which the rank is higher in the case of the overall mobility are areas of concentration of restaurants, malls and leisure centers, where there are workers but their numbers are much smaller than that of visitors.

Finally, to confirm that transportation hubs are more likely to be located in top hotspot levels of the Location History dataset, we calculated how many transit stops fall in the top ranking hotspots in both datasets (Supplementary Table 5). The top level hotspots of the Location History dataset contain almost 700 more stops than the top level commuting hotspots.

Mobility data	Loc. History	Commuting
Stops	2803	2135

Supplementary Table 5: Number of transit stops in top hotspot levels for commuting and Location History data.

Supplementary Note 6: Calculation of Φ from aggregated mobility models

In this section, we investigate if Φ can be effectively reproduced using trip distribution models restricting the analysis to US cities. To do so we will use the Gravity, the Radiation and the Population-weighted opportunities model.

Gravity model

In its simplest formulation, the gravity model assumes the flow of people between areas i and j is proportional to a mass parameter m_i and m_j (which can be the population or the total out/inflow), and inversely proportional to the distance [19, 20]. Mathematically, this can be expressed as

$$T_{ij}^{m} \propto m_i \, m_j \, f(d_{ij}). \tag{3}$$

The deterrence function $f(d_{ij})$ depends only on the distance and it usually takes the form of a power law or an exponential. Here, we will use an exponential decay

$$f(d_{ij}) = e^{-\beta \, d_{ij}}, \tag{4}$$

since it has been shown that it yields better results [21]. The parameter β is fitted from the data.

Radiation model

Despite the wide use of the gravity model, it had several limitations, which led to the development of the radiation model [22]. Here, the main variable is not distance, but the number of job opportunities between two areas i and j. The flow of trips departing from unit i ending in area j can be written as

$$T_{ij}^{m} = m_i \frac{m_i \, m_j}{(m_i + s_{ij})\,(m_i + m_j + s_{ij})}, \tag{5}$$

where m_i and m_j are the masses as in the gravity model and s_{ij} is the number of intervening job opportunities. The standard choice in the radiation model is that the number of jobs is equivalent to the mass.

Population-weighted opportunities model

Nevertheless, the radiation model showed strong discrepancies at intra-urban scales. One proposal to improve the results is the so-called population-weighted opportunities model[23], in which the main variable is the number of job opportunities around the destination instead of the origin and can be written as

$$T_{ij}^{m} = \frac{m_j(\frac{1}{s_{ji}} - \frac{1}{M})}{\sum_{k \neq i}^{N} m_k(\frac{1}{s_{jk}} - \frac{1}{M})}. \tag{6}$$

Here N is the total number of unit areas in the city, M is the total mass of the city and s_{ji} is the number of job opportunities between j and i. As in the radiation model, the job opportunities in every cell is proportional to the mass.

Model comparison

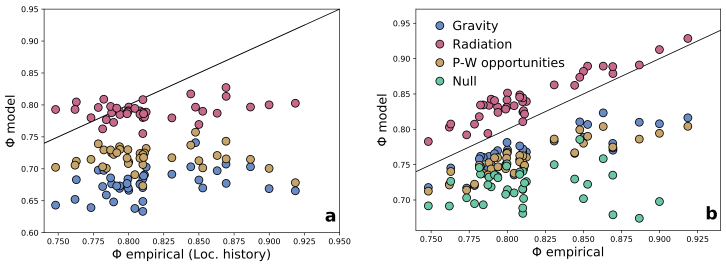

Supplementary Figure 13: Comparison of the flow-hierarchy Φ calculated from trip distribution models. Using **a** the population (total number of commuters) and **b** the outflow as input. The models compared are the gravity, the radiation, the population-weighted opportunities and the null model described in the main manuscript.

We focus here on US cities and evaluate the capability of trip distribution models to reproduce the empirical value of Φ. To do so, we use two different quantities as input for the population m_i of each unit i: the total outflow of commuters obtained from the census, and the same obtained directly from the Location History data. Supplementary Figs. 13**a** and **b** show the comparison obtained from the models for each inputs. In Supplementary Fig. 13**a**, we see that the use of the total number of commuters as input does a poor job of reproducing the empirical values. It is important to note that most of the previous models have been extensively studied for commuting trips at a lower spatial resolution, yet the dataset studied here includes all types of trips.

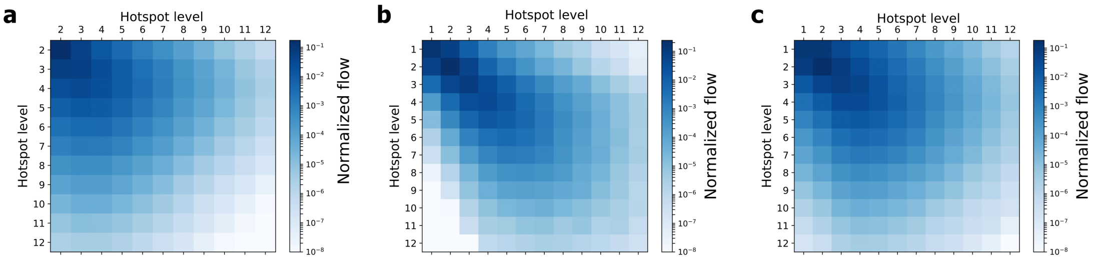

Supplementary Figure 14: Comparison of the matrix of flows between hotspots obtained for each of the trip distribution models using the node outflow as input. The models compared are **a** the gravity, **b** the radiation **c** and the population-weighted opportunities.

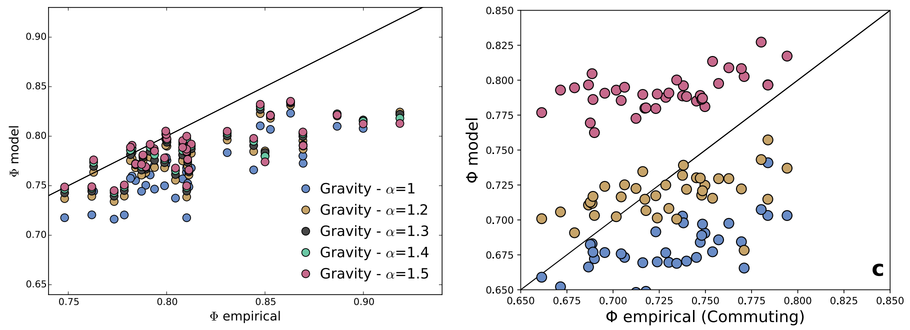

Supplementary Figure 15: Comparison of Φ obtained for the nonlinear gravity model and from the commuting dataComparison of the matrix of flows between hotspots obtained for the nonlinear gravity model. **a** Comparison of the Φ obtained for the nonlinear gravity model and the Location History data.**b** Comparison of the flow-hierarchy Φ for commuting and the equivalent metric calculated from trip distribution models. The models compared are the gravity, the radiation and the population-weighted opportunities.

However, when we use the outflow of Location History as input (Supplementary Fig. 13**a**) we see a much better correlation of the flow-hierarchy between the models and the data, with the radiation model being the most accurate. However, no model exactly reproduces the empirical values. The stronger non-linearities present in the radiation model foster the flows between top hotspot levels leading to a mild overestimation of Φ, while the linear dependence on the masses in the other two models generates comparatively less flows between high-level hotspots thus leading to an underestimation. This can be seen in the matrices of flows between hotspots (Supplementary Fig. 14 for New York City), where the radiation model produces a hierarchical structure that most closely matches the real structure of New York (Figure 3**b**). To confirm this hypothesis we test the gravity model with a non-linearity coefficient α in the population of the destination (Supplementary Fig. 15), finding increasingly good agreement with with empirical values as one moves from a linear to quadratic dependence.

To complete the picture, we run the same analysis but with commuting data alone (Supplementary Fig. 15). The results are similar to those obtained from the Location History.

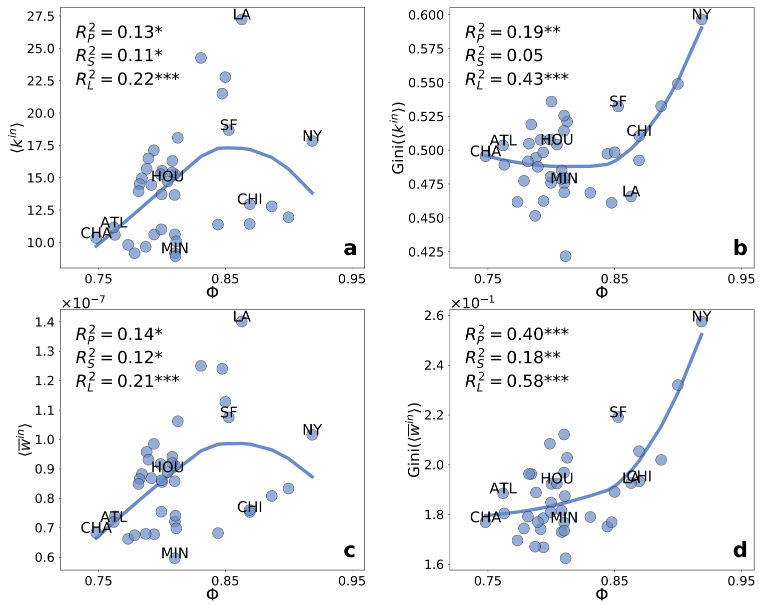

Supplementary Figure 16: **Connecting Φ to population-mixing in US cities**. **a** Average degree $\langle k^{\text{in}} \rangle$ and **b** the Gini coefficient of its distribution as function of Φ. **c** Average weight of incoming links $\langle \overline{w}^{\text{in}} \rangle$ and **d** the Gini coefficient of its distribution. The Gini coefficient of a distribution is given by the area between the Lorenz curve and the diagonal, which corresponds to the Lorenz curve of an homogeneous distribution. Correlations are measured using Pearson, Spearman and LOESS with the corresponding explained variances denoted as R_P^2, R_S^2 and R_L^2, (details in Supplementary Note 9). Asterisks correspond to significance-level of regressions (one * is less than 0.05, two less than 0.01 and three less than 0.001). Some city names appear in the plots: ATL (Atlanta), CHA (Charlotte), CHI (Chicago), HOU (Houston), LA (Los Angeles), MIN (Minneapolis), NY (New York City) and SF (San Francisco).

Supplementary Note 7: Population mixing

We provide here an examination the level of population-mixing in our considered cities. The population-mixing is a particularly relevant measure in social-science applications capturing levels of inequality and accessibility [24, 25]. The metrics proposed for estimating the level of mixing aim at quantifying this effect. A simple measure of mixing is the average incoming degree $\langle k^{\text{in}} \rangle$ of the cells. The stronger the connectivity between cells, the larger the likelihood of urban citizens encountering each other. A related measure is the the average weight of the incoming links for a cell i defined as

$$\overline{w}_i^{\text{in}} = \frac{1}{k_i^{\text{in}}} \sum_{j=0}^{k_i^{\text{in}}} w_{ji}. \qquad (7)$$

The average weight, $\overline{w}_i^{\text{in}}$, can be used to calculate the average over all the cells in the city, $\langle \overline{w}^{\text{in}} \rangle$, and in addition, we also compute the Gini coefficient for both k_i^{in} and $\overline{w}_i^{\text{in}}$ to understand the differences in mixing across the city. In Fig. 16, we show the dependence of these metrics on Φ for US cities, finding a weak but monotonic and statistically significant positive trend with Φ, implying stronger levels of mixing in more hierarchical urban areas. Correlations are measured using Pearson, Spearman and LOESS with the corresponding variances denoted as R_P^2, R_S^2 and R_L^2 (Details in Supplementary Note 9). Similar results are obtained when a more involved mixing metric based on entropy measures is applied (see Supplementary Note 7 and Supplementary Fig. 16). The Gini coefficients indicate that for cities with larger Φ, the mixing is on average more concentrated due to the more intense flows of hotspots at the same level. While the stronger connections between hotspots facilitate population-mixing, we note that factors such as geography and transportation infrastructure are likely to play a role.

We complete the previous results with a more elaborate metric, which aims at capturing the difference of the incoming mobility flows in every cell i. Defining w_{ji} as the trip flow from cell j to i, we normalize

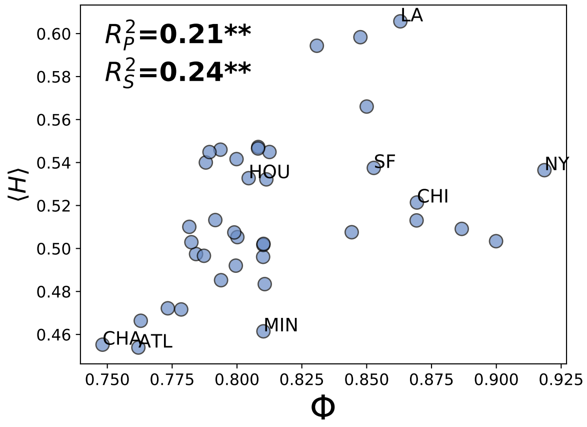

Supplementary Figure 17: Population-mixing in US cities as a function of hierarchical structure. Average flow entropy $\langle H \rangle$ as a function of Φ.

it by the total outflow of j, f_j^{out}, thus,

$$\rho_{ji} = \frac{w_{ji}}{f_j^{out}}. \tag{8}$$

The quantity ρ_{ji} represents the fraction of trips starting in j and ending in i. Normalizing again with respect to the trip-sources j,

$$p_{ji} = \frac{\rho_{ji}}{\sum_k \rho_{ki}}, \tag{9}$$

yields a measure of the relevance of j as a source of trips to i. Next, we define an entropy H_i for every cell i,

$$H_i = \frac{\sum_{j=1}^{k_i^{in}} p_{ji}\, ln(p_{ji})}{ln(k_i^{in})}, \tag{10}$$

where k_i^{in} is the in-degree of i. The entropy can be averaged over cells to obtain $\langle H \rangle$. In Supplementary Fig. 17, we show $\langle H \rangle$ as a function of Φ. The picture is more or less the same than the one provided in the main manuscript, corroborating weak but statistically significant correlation between levels of mixing and and increasing hierarchy as measured by Φ.

Supplementary Note 8: Metadata

Modal share of transport, emission of pollutants and health indicators for US cities

- The modal share of commuting trips for all the US metropolitan areas was sourced from the census website [26], which provides the percentage of commuting trips in terms of transportation mode.
- The smoking rate by city was obtained from [27].
- Pollutant emissions were obtained from the United States Environmental Protection Agency (US EPA) which makes public a National Emissions Inventory (NEI) every three years. We used the version corresponding to 2014 [28]. This inventory is at the scale of counties, which requires merging them at the level of Metropolitan-Micropolitan areas. The total emissions for each metropolitan area were calculated as the sum of emissions of the counties inside the metropolitan areas, and then dividing it by the total population, to obtain the emissions per capita. The information about which counties are included in each metropolitan area is available in [29].
- The incidence of ischemic stroke and all types of stroke, including morbidity and mortality, has been obtained at the scale of counties was sourced from the Centers for Disease Control and Prevention

(CDC) of the United States [30]. The aggregation procedure used is similar to the one used for the emissions. The prevalence (cases per 10^4 individuals) was converted to total cases per county. This was then aggregated for all counties in a metropolitan area and then divided by the population. Obesity has been obtained from the same source and followed the same aggregation procedure.

- Transport and health indicators related to traffic fatal injuries have been obtained from [31].
- General mortality and mortality by age were obtained from [32].
- Mortality by chronic obstructive pulmonary disease was obtained from [33].
- Data about the location of acute care hospitals in the United States was obtained from [34].

Modal share of transport and pollutant emissions for global cities

- Modal share of transport obtained from [35].
- Greenhouse gas (GHG) emissions were obtained from the Climate Disclosure Project (CDP) [36] where a set of worldwide cities make public their annual emissions in equivalent carbon dioxide metric tones per capita.
- The OECD also reports CO_2 emissions per capita within their defined boundaries for a large number of cities [37] .
- Emissions of other pollutants, including NO_x, obtained from the Emissions Database for Global Atmospheric Research [38], which provides a grid map of emissions of a set pollutants. The total emissions for each city were computed by summing up all the cells included in the city boundaries and dividing by the population to obtain per capita emissions. The dataset and procedure is similar to that used in [39]

Supplementary Note 9: Robustness of correlations

Metrics

Three types of correlation coefficients were used in this analysis. The first is Pearson's r, which measures the linear correlation between two variables, and is calculated as

$$r_{x,y} = \frac{cov(x,y)}{\sigma_x \sigma_y}, \tag{11}$$

where cov is the covariance, and σ_x (σ_y) is the standard deviation of the samples x (y). In a linear regression model, the square of the Pearson correlation is the fraction of explained variance. We denote the square of the Pearson correlation coefficient as R_P^2.

The second is the non parametric Spearman correlation ρ based on rank l. Unlike the Pearson coefficient, the Spearman is not necessarily linear as it evaluates the monotonic relation between two variables. The formula is identical to that for r, with the replacement of x and y by their ranks l_x and l_y,

$$\rho_{x,y} = \frac{cov(l_x,l_y)}{\sigma_{l_x} \sigma_{l_y}}, \tag{12}$$

We will denote the the square of the Spearman correlation coefficient as R_S^2.

Finally, we employ a polynomial regression based on LOESS (locally estimated scatterplot smoothing) [40, 41, 42, 43], which is a nonlinear piecewise regression; instead of fitting a concrete function it fits a spline. We will denote this as R_L^2. The p-values obtained with the LOESS regression are computed using a F-test statistic thus,

$$F = \frac{(TSS_y - RSS_{LOESS})/(df_{LOESS} - 1)}{(RSS_{LOESS})/(N - df_{LOESS})}, \tag{13}$$

where TSS_y is the total sum of squares of the dependent variable, RSS is the residual sum of squares obtained from the LOESS regression, N is the number of observations and df are the degrees of freedom of the LOESS regression based on the equivalent number of parameters [44].

US cities

We show here additional results for US cities complementing Fig. 3 in the main manuscript.

p-values for correlations reported in manuscript

Supplementary Table 6 shows the p-values obtained with the three regression techniques for the correlations denoted in Fig. 3. The p-values are all below 10^{-3} indicated by three stars in the R^2 values. Metropolitan and micropolitan areas were filtered by population; only those cities with population > 1.4 million are shown. The sensitivity of the correlations to population threshold are indicated in Supplementary Table 7 with thresholds varying between from 400000 to 2.6 million inhabitants. p-values show that all indicators have a certain level of significance (<0.01) for all population thresholds below 2.6 million. In general the larger the cities, the stronger the correlations. We also tested the effect of removing New York City from Fig. 3. Despite the slight drop in the correlations, it is only appreciable in the modal shares by car and public transportation (Supplementary Fig. 18).

Method	Drive M.Share	PT M.share	Walk M.Share	NOx Emissions	CO Emissions	PM10 Emissions	Isch. Stroke mort.	Hosp. Dist.	Traff. Injur.
Pearson	1.93e-05	4.11e-06	1.6e-06	2.01e-06	6.61e-05	6.95e-05	0.000288	1.48e-05	0.000117
Spearman	0.000231	8.03e-06	0.000261	8.78e-07	7.68e-06	0.000105	0.000996	2.64e-05	0.000361
LOESS	4.869e-07	9.31e-09	1.68e-07	2.56e-06	0.000147	2.28e-05	0.000341	2.25e-05	0.000899

Supplementary Table 6: Significance (p-values) for the correlations shown in Fig. 3.

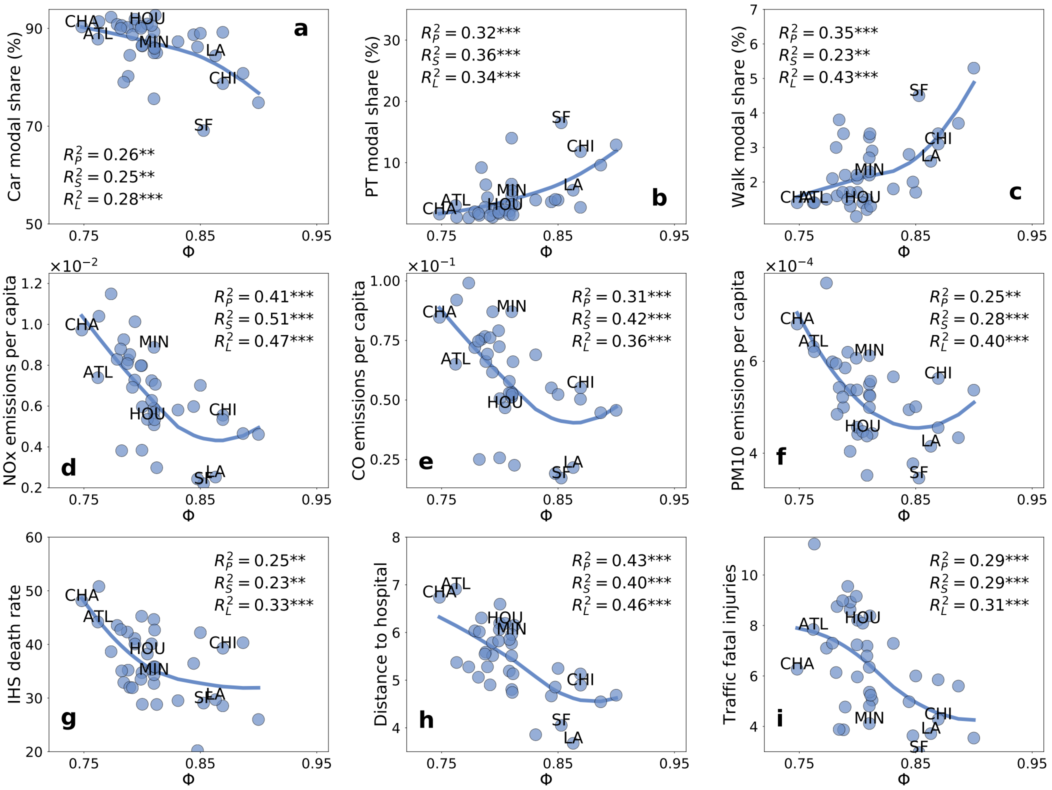

Supplementary Figure 18: (a-c) Modal share for trips to work (%) as a function of the city flow-hierarchy: (a) Modal share by private car, (b) by public transport (PT) and (c) by walk. (d-f) Relation between the flow-hierarchy and the emission of pollutants: (d) NOx, (e) CO and (f) PM10 emissions in metric tons per capita. (g-i) Flow-hierarchy and health: (g) Ischemic stroke mortality in cases per 100,000 inhabitants, (h) Average distance to closest hospital and (i) Incidence of traffic fatal injuries per 100,000 residents.

Population	Drive M.Share	PT M.share	Walk M.Share	CO Emissions	NOx Emissions	PM10 Emissions	PM25 Emissions	Isch. Stroke mort.	Hosp. Dist.	Traff. Injur.
400000	-0.37***	0.42***	0.50***	-0.49***	-0.48***	-0.28**	-0.43***	-0.24**	0.41***	-0.31***
800000	-0.51***	0.54***	0.61***	-0.64***	-0.59***	-0.53***	-0.60***	-0.39**	0.50***	-0.37**
1000000	-0.58***	0.63***	0.67***	-0.61***	-0.54***	-0.51***	-0.59***	-0.43**	0.60***	-0.48***
1200000	-0.60***	0.64***	0.67***	-0.63***	-0.56***	-0.50***	-0.59***	-0.44**	0.61***	-0.51***
1400000	-0.63***	0.67***	0.68***	-0.69***	-0.60***	-0.51***	-0.59***	-0.55***	0.64***	-0.58***
1800000	-0.68***	0.71***	0.69***	-0.66***	-0.57***	-0.52**	-0.56***	-0.51**	0.68***	-0.56***
2000000	-0.71***	0.74***	0.73***	-0.66***	-0.54**	-0.47**	-0.53**	-0.55**	0.71***	-0.55**
2200000	-0.69***	0.72***	0.72***	-0.69***	-0.56**	-0.49**	-0.57**	-0.53**	0.70***	-0.53**
2600000	-0.71***	0.73***	0.76***	-0.62**	-0.47**	-0.41	-0.48**	-0.50**	0.72***	-0.66**

Supplementary Table 7: Correlations between flow-hierarchy and modal share, pollution and health measures for different population thresholds. * p-value<0.05 , ** p-value<0.01,*** p-value<0.001

Temporal evolution of correlations

Next, we analyze the temporal evolution of the correlation of Φ with urban indicators. We aggregated the weekly networks into months, to test the stability of correlations in time. Supplementary Table 8, indicates that, apart from some slight variations, both r and the corresponding p-values are stable at the monthly level.

Year	Month	Drive M.Share	PT M.share	Walk M.Share	CO Emissions	NOx Emissions	PM10 Emissions	PM25 Emissions	Isch. Stroke mort.	Traff. Injur.	Hosp. Dist.
2016	0	-0.56***	0.61***	0.62***	-0.67***	-0.58***	-0.50**	-0.56***	-0.51***	-0.51***	-0.64***
2016	1	-0.58***	0.62***	0.63***	-0.70***	-0.63***	-0.50**	-0.61***	-0.51***	-0.55***	-0.63***
2016	2	-0.58***	0.63***	0.66***	-0.71***	-0.62***	-0.51***	-0.60***	-0.53***	-0.56***	-0.63***
2016	3	-0.61***	0.65***	0.68***	-0.69***	-0.60***	-0.53***	-0.61***	-0.50**	-0.55***	-0.64***
2016	4	-0.61***	0.66***	0.70***	-0.66***	-0.57***	-0.42**	-0.53***	-0.49**	-0.59***	-0.65***
2016	5	-0.61***	0.66***	0.69***	-0.67***	-0.58***	-0.47**	-0.56***	-0.53***	-0.59***	-0.64***
2016	6	-0.62***	0.66***	0.67***	-0.68***	-0.59***	-0.46**	-0.57***	-0.58***	-0.63***	-0.61***
2016	7	-0.61***	0.65***	0.64***	-0.67***	-0.59***	-0.48**	-0.58***	-0.58***	-0.62***	-0.60***
2016	8	-0.63***	0.67***	0.65***	-0.71***	-0.63***	-0.53***	-0.62***	-0.56***	-0.60***	-0.61***
2016	9	-0.67***	0.70***	0.71***	-0.72***	-0.64***	-0.57***	-0.62***	-0.56***	-0.61***	-0.62***
2016	10	-0.66***	0.69***	0.72***	-0.68***	-0.61***	-0.55***	-0.58***	-0.51***	-0.54***	-0.64***
2016	11	-0.67***	0.69***	0.72***	-0.68***	-0.61***	-0.57***	-0.61***	-0.53***	-0.56***	-0.64***
2016	12	-0.68***	0.69***	0.73***	-0.71***	-0.64***	-0.58***	-0.62***	-0.58***	-0.54***	-0.63***

Supplementary Table 8: Temporal evolution of the correlations between Φ and modal share, pollution and health measures. The weekly networks are aggregated into groups of four weeks. The population threshold used is 1400000 * p-value<0.05 , ** p-value<0.01,*** p-value<0.001

Connection to other transportation metrics

The relationship between Φ and the use of different transport modes is studied through the modal share of commuting trips. Commuting, however, may not be representative of the global mobility of cities and thus we also check the correlation with other sources of data related to transport modalities. The Bureau of Transport Statistics (BTS) [31], provides transport data on cities including Vehicle Miles Traveled (VMT) per capita (including length of trips) and transit trips per capita which include more than merely commuting trips. Supplementary Fig. 19 shows the relation between both metrics and Φ, indicating a high correlation. Similar to that seen for modal share, the VMT is anti-correlated with Φ, with a higher number of transit trips and a smaller number of VMT per capita in hierarchical cities, and the opposite in those with less hierarchical structure.

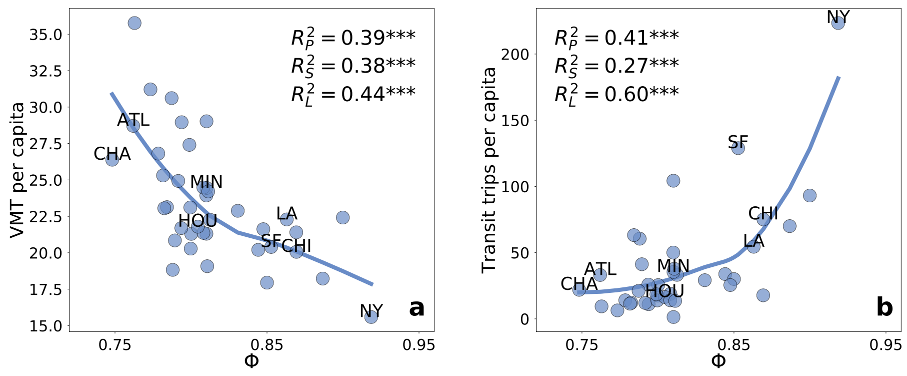

Supplementary Figure 19: Relation between Φ and **a** vehicle miles traveled, and **b** transit trips per capita

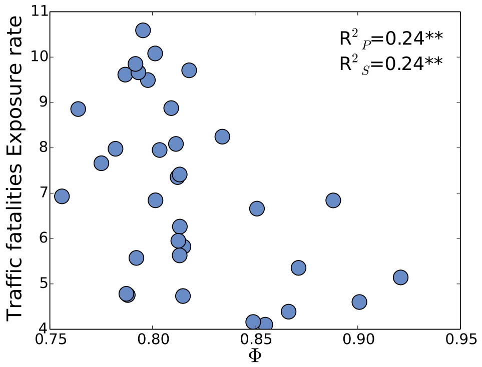

Supplementary Figure 20: Relation between Φ and exposure rate of traffic fatal injuries.

It is also important to complement the correlation of Φ and traffic fatalities, with the exposure ratio, also available from the Bureau of Transport. The exposure ratio modifies the number of fatal injuries by accounting for modal share. This corrects for the influence of preferred modal share in each city. As is seen in Supplementary Fig. 20, despite a slightly weaker correlation, the statistical significance of the relation is intact.

Relating ischemic stroke to emergency service access

In Fig. 3 we report a strong correlation between the death rate from ischemic stroke and Φ. A possible reason for this connection might be the degree of accessibility to emergency services. We test this hypothesis by examining the prevalence of ischemic stroke and the rate of survival. In Supplementary Fig. 21 **a** we plot the incidence by ischemic stroke against the flow-hierarchy showing that the correlation is significantly lower than the displayed with mortality. Thereby, the accessibility to public health facilities –correlated with the flow-hierarchy– plays a role in the mortality by ischemic stroke. Moreover, if we plot the ischemic stroke survival rate (Supplementary Fig. 21 **b**) calculated as one minus the rate between individuals deceased and survivors the correlation increases again despite one outlier. Overall, those cities with a higher flow-hierarchy display a higher rate of survivors from ischemic stroke.

Comparison with population density

We observe that US cities with a higher value of Φ have also high population densities (values for each CBSA obtained from census [26]). Supplementary Fig. 23 shows the population density as a function of Φ; while there is a positive correlation, the density accounts for only a fraction of the information contained in Φ ($R^2_P = 0.52$). Indeed, as marked in the figure, New York and Los Angeles have comparable

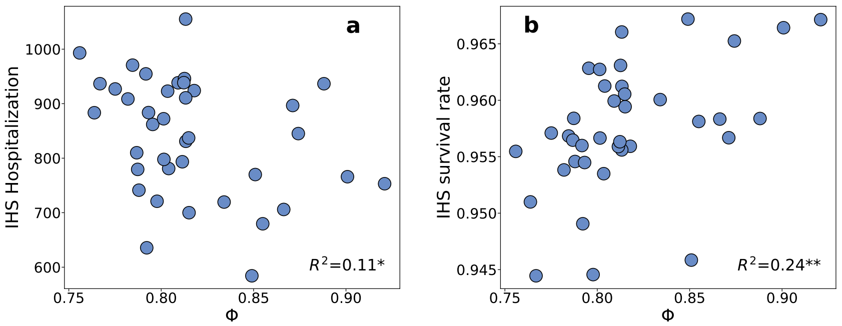

Supplementary Figure 21: Relation between Φ and **a** the incidence of ischemic stroke; **b** the rate of survival from ischemic stroke;

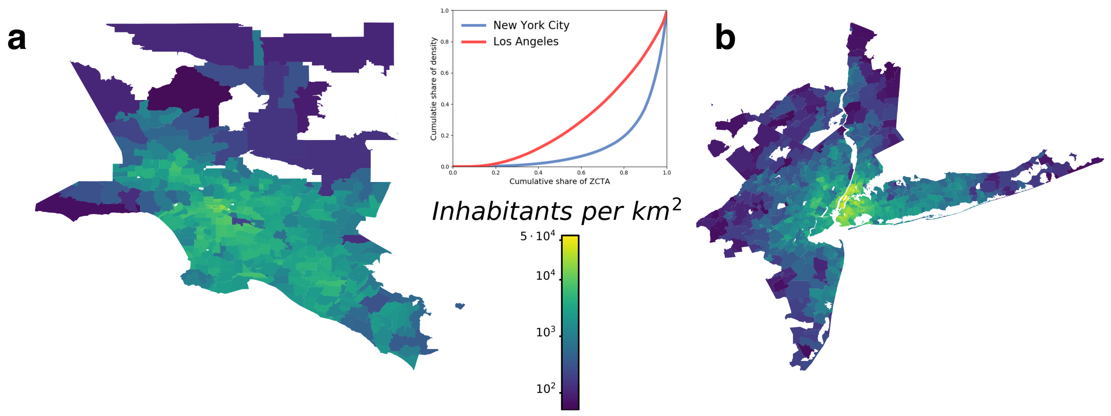

Supplementary Figure 22: Comparison between the spatial distribution of population density in Los Angeles and New York City. The top inset shows the Lorenz curve of the distribution of population density at the level of ZCTA. Bearing in mind that the diagonal corresponds to an homogeneous even distribution, it is clear that the distribution of population density in New York City is much more unequal. The map of population densities also correspond to **a** Los Angeles and **b** New York. Again while a few areas reach the maximum value in New York, in Los Angeles they do not even reach half of it, displaying a more homogeneous map

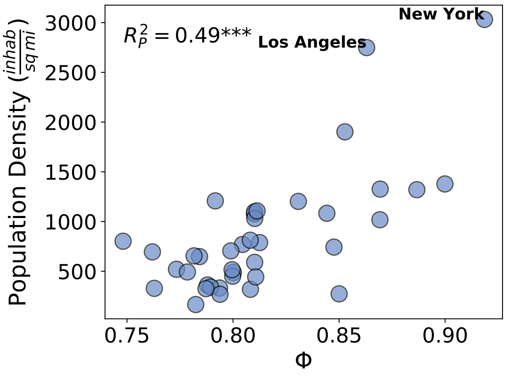

Supplementary Figure 23: Relation between Φ and population density.

average population densities, but different values of Φ. Φ appears to capture the spatial differences in the population densities; as depicted in Supplementary Fig. 22, Los Angeles has a more homogeneous density, while New York has a strong peak in the core of the city (a feature captured by Φ and not found in the raw density values).

Previous work has examined the connection between population density, mode of transport , emission of pollutants and traffic fatal injuries [45, 46, 47, 48, 49]. We repeat the analysis shown in Fig. 3, but now using the population density as shown in Supplementary Fig. 24. While the correlations with the modal share of commuting, and the average distance to the closes hospital [52], is comparable to that seen for Φ, there is a significant decrease seen for emission of pollutants and health indicators. Indeed, Φ displays a higher ρ for all urban indicators and a higher r in 7 of the 9 urban indicators. In addition, the regression with Φ also has higher statistical significance (p-values<0.001) for all urban indicators. The flow-hierarchy thus contains significantly more information about the city than the population density.

Comparison with urban sprawl

The study of urban sprawl has been an active field among researchers, focusing on its quantification of and consequences on health, environment, transport, and segregation among others. A summary of the

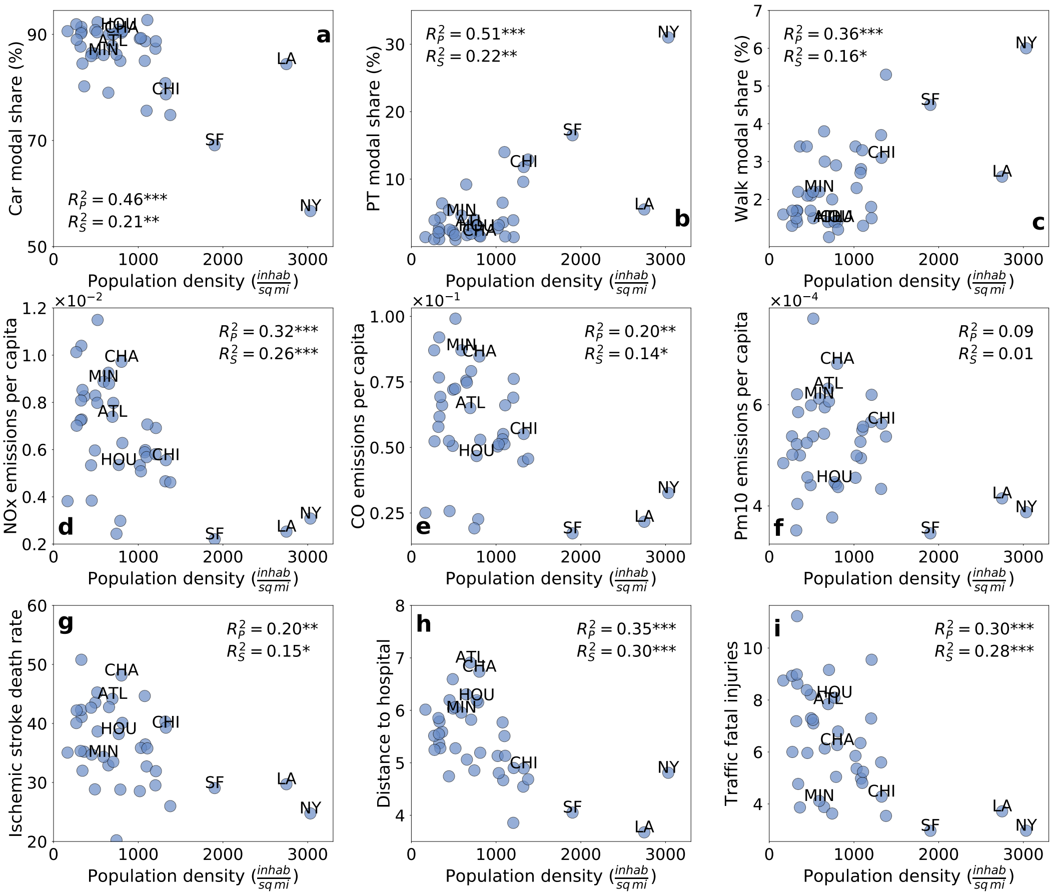

Supplementary Figure 24: Results for US cities. Relation between the population density and modal split, pollution and health. **a-c** Modal share for trips to work (%) as a function of the city trace: **a** Modal share by private car, **b** by public transport and **c** by walk. **d-f** Relation between city density and emission of pollutants: **d** NO_X, **e** CO and **f** PM_{10} emissions in metric tons per capita. **g-i** Relation between city density and health: **g** Death rate of ischemic stroke in cases per 100000 inhabitants, **h** Average distance to closest hospital and **i** Incidence of traffic fatal injuries per 100000 residents (* p-value<0.05 , ** p-value<0.01,*** p-value<0.001). Some city names appear in the plots: ATL (Atlanta), CHA (Charlotte), CHI Chicago), HOU (Houston), LA (Los Angeles), MIN (Minnesota), NY (New York City) and SF (San Francisco).

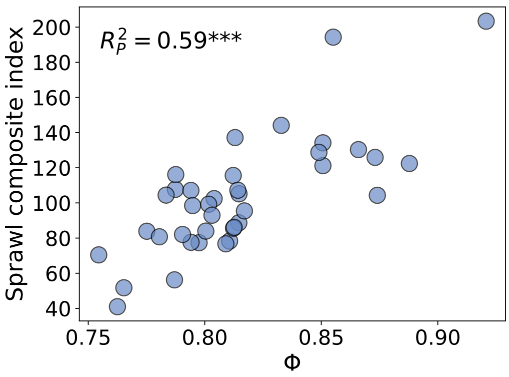

Supplementary Figure 25: Relation between Φ and the sprawl composite index

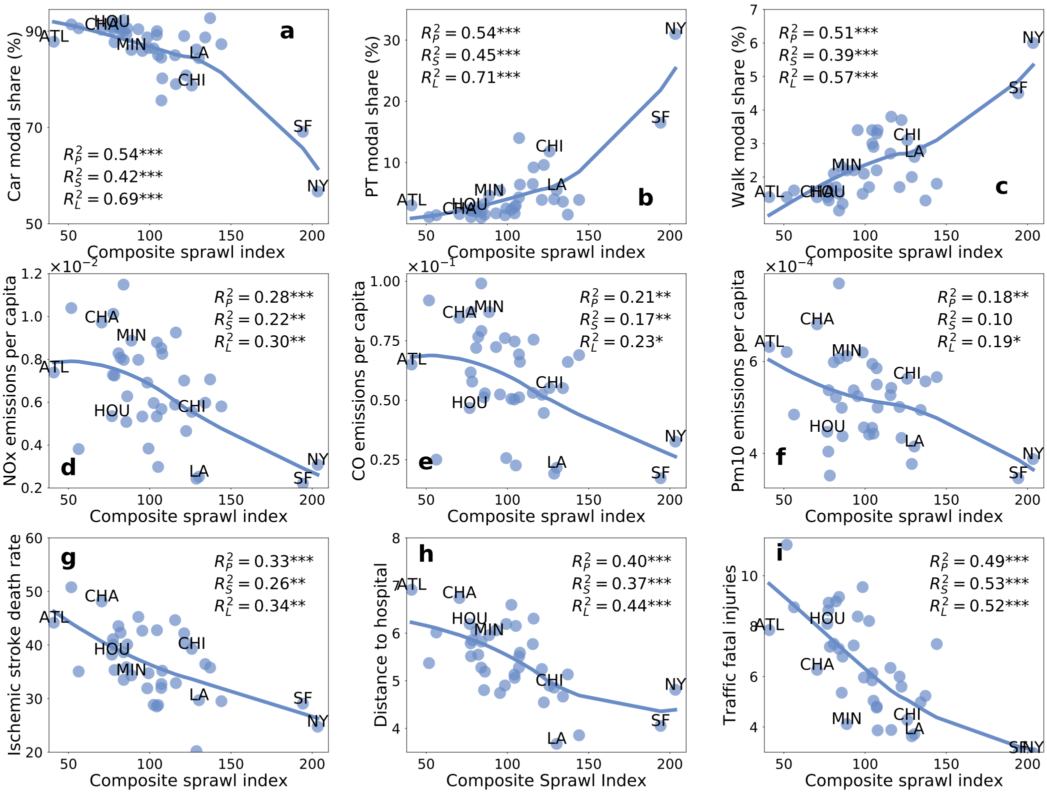

Supplementary Figure 26: Results for US cities. Relation between the sprawl composite index and modal split, pollution and health. **a-c** Modal share for trips to work (%) as a function of the city sprawl composite index: **a** Modal share by private car, **b** by public transport and **c** by walk. **d-f** Relation between the sprawl composite index and emission of pollutants: **d** NO_X, **e** CO and **f** PM_{10} emissions in metric tons per capita. **g-i** Sprawl composite index and health: **g** Death rate of ischemic stroke in cases per 100000 inhabitants, **h** Average distance to closest hospital and **i** Incidence of traffic fatal injuries per 100000 residents (* p-value<0.05 , ** p-value<0.01,*** p-value<0.001). Some city names appear in the plots: ATL (Atlanta), CHA (Charlotte), CHI Chicago), HOU (Houston), LA (Los Angeles), MIN (Minnesota), NY (New York City) and SF (San Francisco).

main findings, including the quantification, causes and consequences of urban sprawl, can be found in [50]. One of the drawbacks of computing urban sprawl is the need to gather multidimensional indices including measures of population density and its gradient, the density of jobs, the mix of land use and the topology of the road network. Keeping in mind this work aims to measure the hierarchical structure of cities and not their sprawl, it is worth investigating the relation between sprawl indices and Φ, given the latter's observed connection with population density. One recent measure of sprawl proposed in [51] combines density of population, mix of land use, strength of activity centers and accessibility of road networks. Each of these features aggregate by itself several indicators so that the final composite index includes 22 different variables.

In Supplementary Fig. 25, we plot the composite index in function of Φ finding a monotonically increasing trend. This suggests that more compact cities are more hierarchical on average, although the relation is far from deterministic given $R_P^2 = 0.58$. The effects of urban sprawl in transport, emission of pollutants and health have been studied in several works [50], and here we assess how its correlation with the urban indicators studied in this work. In Supplementary Fig. 26, we plot the composite index against the measures studied in Fig. 3 of the main text. In most cases the correlations are comparable, with slightly better results for the composite index in the modal split but better results for the flow-hierarchy in emission indices. Similarly The p-values obtained (Supplementary Table 9) are similar to those obtained for Φ (Supplementary Table 6). While both indicators contain similar information on transport, emission of pollutants and health in cities, the advantage afforded by Φ is that it is directly

measurable from mobility data, without the need to aggregate 22 variables. Furthermore, it can be easily updated in time.

Method	Drive M.Share	PT M.share	Walk M.Share	NOx Emissions	CO Emissions	PM10 Emissions	Isch. Stroke mort. mortality	Hosp. Dist.	Traff. Injur.
Pearson	1.32e-07	1.51e-07	4.35e-07	0.000595	0.00342	0.00704	0.000161	2.25e-05	9.45e-07
Spearman	1.22e-05	4.67e-06	3.11e-05	0.00339	0.00974	0.0526	0.00118	6.4e-05	8.45e-07
LOESS	8.242e-10	2.3e-10	4.11e-07	0.00347	0.0203	0.0442	0.00108	4.54e-05	3e-06

Supplementary Table 9: Significance (p-values) for the correlations shown in Supplementary Fig. 26.

Multivariate analysis of flow hierarchy with socioeconomic and behavioral factors

We have thus far, examined the direct relationship between Φ and the urban indicators, yet the latter might also be influenced by other socioeconomic variables and behavioral factors. To check for this, we conduct a multivariate analysis of Φ with health, environmental and modal share indicators, taking into account other variables that might be relevant to test its significance.

We start with the modal share of transport. Reasonable choices for factors influencing this are the GDP per capita and the percentage of poverty. While the GDP captures the overall economic situation of a city, the percentage of poverty mirrors economic inequality. Our analysis indicates that the percentage of poverty turned out to be statistically insignificant. The results for GDP and Φ are shown for the modal share by car, public transportation and walking in Supplementary Tables 10, 11 and Table 12 respectively. In the modal share by car, both GDP and the Φ are highly significant (p-value<0.001) and explain together half of the total variance. In the case of modal share by public transportation and walking, Φ is more significant than the GDP.

Next we examine the emission of pollutants, again using the GDP per capita and the poverty-percentage as additional variables. In contrast to transport, poverty is significant for emissions. The multivariate analysis for the emissions of CO, NO_x,$PM10$ and $PM25$ are shown in Supplementary Tables 13, 14, 15 and 16 respectively. The flow-hierarchy Φ is significantly correlated with the emissions of all the pollutants even when considering economic variables, among which the poverty-percentage is the most correlated. For PM10 and PM25 emissions, the GDP per capita appears to be uncorrelated.

As shown in the main manuscript, Φ is connected to health indicators. It is thus worth examining what the extent of this connection is when accounting for other standard variables that are related to health. We start by checking if the distance to the closest hospital, as discussed in the main text, is influenced by the economic situation of cities. Supplementary Table 17 indicates no discernible connection between economic variables with the spatial distribution of healthcare facilities. For spatial distribution of hospitals or the walkability of cities, we check the influence of the percentage of the population that engages in binge drinking, the smoking rate, the percentage of population with obesity and economic variables, the GDP per capita and the percentage of poverty. In all cases we perform a first analysis with all the variables and discard some of them depending on the correlation obtained and its significance. We start the analysis with the most general health indicator, mortality, specifically, age dependent mortality, observing a correlation with the mortality between 0 and 5 years old and between 5 and 25 when other possible variables related taken into account.

Other variables that are correlated according tare the smoking percentage in the mortality between 0 and 5 years old, and the GDP per capita in the case of ages between 5 and 25 years. We show in Supplementary Tables 18 and 20 the analysis when all variables are considered for the mortality between 0 and 5 and between 5 and 25 respectively, and in Supplementary Tables 19 and 21 the final analysis considering only the most significant ones in each case. While the smoking percentage can be relevant in the early stages of live, as kids are more susceptible to smoke in the environment, it is not so relevant as they grow up and become less sensible to the smoke. On the contrary, as kids grow up, the leading causes of death are, among others, suicide and homicide, which are susceptible of being related to economic variables such as the GDP per capita. When controlling that variables, the flow-hierarchy appears to be significant in both cases. While in the window between 0 and 5 years old, the flow-hierarchy explains 10% of the variance explained by smoking, it explains 9% of the total variance of mortality between 5 and 25 years old.

Beyond the overall mortality, we also find correlations with other causes of death, likely a consequence of the better accessibility to healthcare facilities. Supplementary Tables 22 and 23 show the multivariate analysis for the mortal Chronic obstructive pulmonary disease (COPD) mortality when considering all

possible variables and only the most significant respectively. As one could expect, the other relevant variable apart from the flow-hierarchy is smoking rate. Despite the high correlation with the smoking rate, the flow-hierarchy also explains part of the variance, concretely more than one third of the variance explained by the smoking rate. In Supplementary Tables 24 and 25 we show the analysis of the mortality by all type of strokes when considering all variables or only the most relevant ones. As it can be seen, the flow-hierarchy is also correlated when controlling other variables, explaining one fourth of the total variance.

Similarly, we show in Supplementary Tables 26 and 27 the multivariate analysis for the ischemic stroke mortality, in which the flow-hierarchy is higher equally significant, and explains 10% of the total variance. Interestingly more, the correlation with the ischemic stroke only appears in the death rate, being not correlated with the hospitalization as can be seen in Supplementary Table 28. This reinforces the connection between the death rate and the accessibility to hospitals, as the correlation is not with the prevalence but with the survival. Finally we perform a multivariate analysis with the mortality by transport fatalities, showing that the flow-hierarchy together with the GDP are significantly correlated with it. Apart from the effect of an easier access to healthcare facilities, the modal share in cities also plays a role in this case. As can be seen in Supplementary Tables 30 and 29, the flow-hierarchy explains one fifth of the total variance of the mortality by fatal injuries in any mode of transport.

Supplementary Table 10: Multivariate analysis of the modal share by car

Variable	Sum Sq	Variance	Df	F value	P-value
Flow-hierarchy	320.0161	0.216	1.0	14.946	0.000445***
GDP	389.2594	0.263	1.0	18.180	0.000139***
Residuals	770.8138	0.521	36.0		

*p<0.1; **p<0.05; ***p<0.01

Supplementary Table 11: Multivariate analysis of the modal share by public transportation

Variable	Sum Sq	Variance	Df	F value	P-value
GDP	176.2129	0.183	1.0	12.326	0.001222**
Flow-hierarchy	273.7042	0.284	1.0	19.145	0.000099***
Residuals	514.6638	0.534	36.0		

*p<0.1; **p<0.05; ***p<0.01

Supplementary Table 12: Multivariate analysis of the modal share by walking

Variable	Sum Sq	Variance	Df	F value	P-value
Flow-hierarchy	11.6408	0.303	1.0	20.984	0.000054***
GDP	6.8497	0.178	1.0	12.348	0.001211**
Residuals	19.9705	0.519	36.0		

*p<0.1; **p<0.05; ***p<0.01

In Tables 24 and 25 we show the analysis of the mortality by all type of strokes when considering all variables or only the most relevant ones. As it can be seen, the flow-hierarchy is also correlated when controlling other variables, explaining one fourth of the total variance.

Supplementary Table 13: Multivariate analysis of the Emissions of CO

Variable	Sum Sq	Variance	Df	F value	P-value
GDP	3.214E-03	0.208	1.0	13.960	0.000664***
Perc. of poverty	3.161E-03	0.205	1.0	13.732	0.000725***
Flow-hierarchy	9.971E-04	0.065	1.0	4.331	0.044803*
Residuals	8.058E-03	0.522	35.0		

*p<0.1; **p<0.05; ***p<0.01

Supplementary Table 14: Multivariate analysis of the Emissions of NO_x

Variable	Sum Sq	Variance	Df	F value	P-value
Flow-hierarchy	2.296E-05	0.134	1.0	10.341	0.002799**
GDP	3.449E-05	0.201	1.0	15.533	0.000370***
Perc. of poverty	3.620E-05	0.211	1.0	16.305	0.000280***
Residuals	7.771E-05	0.453	35.0		

*p<0.1; **p<0.05; ***p<0.01

Supplementary Table 15: Multivariate analysis of the Emissions of $PM10$

Variable	Sum Sq	Variance	Df	F value	P-value
Flow-hierarchy	2.481E-08	0.086	1.0	4.470	0.041687*
GDP	3.246E-08	0.113	1.0	5.847	0.020941*
Perc. of poverty	3.651E-08	0.127	1.0	6.579	0.014767*
Residuals	1.943E-07	0.674	35.0		

*p<0.1; **p<0.05; ***p<0.01

Supplementary Table 16: Multivariate analysis of the Emissions of $PM25$

Variable	Sum Sq	Variance	Df	F value	P-value
Flow-hierarchy	4.953E-09	0.072	1.0	3.565	0.067324
GDP	4.908E-09	0.072	1.0	3.532	0.068532
Perc. of poverty	1.007E-08	0.147	1.0	7.249	0.010812
Residuals	4.863E-08	0.709	35.0		

*p<0.1; **p<0.05; ***p<0.01

Supplementary Table 17: Multivariate analysis of the average distance to the closest hospital

Variable	Sum Sq	Variance	Df	F value	P-value
Flow-hierarchy	6.482E+00	0.337	1.0	17.823	0.000164
GDP	1.768E-02	0.001	1.0	0.049	0.826771
Perc. of poverty	1.045E-03	0.000	1.0	0.003	0.957566
Residuals	1.273E+01	0.662	35.0		

*p<0.1; **p<0.05; ***p<0.01

Supplementary Table 18: Mortality age 0-5 (all variables)

Variable	Sum Sq	Variance	Df	F value	P-value
Flow-hierarchy	6.681E-03	0.046	1.0	2.230	0.146562
Obesity	1.805E-02	0.124	1.0	6.023	0.020598*
GDP	9.333E-05	0.001	1.0	0.031	0.861183
Perc. of poverty	2.584E-03	0.018	1.0	0.863	0.360966
Smoking	2.228E-02	0.153	1.0	7.437	0.010902*
Alcohol	4.005E-03	0.027	1.0	1.337	0.257390
O_3 concen.	1.516E-03	0.010	1.0	0.506	0.482782
CO concen.	6.581E-03	0.045	1.0	2.196	0.149522
NO concen.	4.890E-05	0.000	1.0	0.016	0.899257
SO concen.	1.288E-05	0.000	1.0	0.004	0.948184
Residuals	8.390E-02	0.576	28.0		

*p<0.1; **p<0.05; ***p<0.01

Supplementary Table 19: Mortality age 0-5 (significant variables)

Variable	Sum Sq	Variance	Df	F value	P-value
Flow-hierarchy	2.400E-02	0.069	1.0	6.082	0.018550*
Smoking	1.809E-01	0.521	1.0	45.850	6.6E-08***
Residuals	1.421E-01	0.409	36.0		

*p<0.1; **p<0.05; ***p<0.01

Supplementary Table 20: Mortality age 5-25 (all variables)

Variable	Sum Sq	Variance	Df	F value	P-value
Flow-hierarchy	3.216E-02	0.117	1.0	4.832	0.036364*
Obesity	8.740E-03	0.032	1.0	1.313	0.261507
GDP	1.874E-03	0.007	1.0	0.282	0.599865
Perc. of poverty	6.529E-03	0.024	1.0	0.981	0.330422
Smoking	4.332E-03	0.016	1.0	0.651	0.426558
Alcohol	9.499E-03	0.035	1.0	1.427	0.242224
O_3 concen.	8.777E-04	0.003	1.0	0.132	0.719215
CO concen.	1.107E-02	0.040	1.0	1.663	0.207707
NO concen.	1.074E-02	0.039	1.0	1.614	0.214413
SO concen.	1.646E-03	0.006	1.0	0.247	0.622811
Residuals	1.863E-01	0.681	28.0		

*p<0.1; **p<0.05; ***p<0.01

Supplementary Table 21: Mortality age 5-25 (significant variables)

Variable	Sum Sq	Variance	Df	F value	P-value
GDP	1.492E-01	0.326	1.0	19.921	0.000076***
Flow-hierarchy	3.943E-02	0.086	1.0	5.264	0.027711*
Residuals	2.696E-01	0.588	36.0		

*p<0.1; **p<0.05; ***p<0.01

Supplementary Table 22: Mortality by COPD (all variables)

Variable	Sum Sq	Variance	Df	F value	P-value
Flow-hierarchy	7.611E+01	0.024	1.0	1.234	0.276045
Obesity	1.676E+01	0.005	1.0	0.272	0.606206
GDP	3.235E+02	0.100	1.0	5.246	0.029734*
Perc. of poverty	1.957E+02	0.060	1.0	3.173	0.085738
Smoking	6.285E+02	0.194	1.0	10.192	0.003471**
Alcohol	1.161E+02	0.036	1.0	1.882	0.181005
O_3 concen.	6.106E+00	0.002	1.0	0.099	0.755345
SO concen.	7.335E+01	0.023	1.0	1.189	0.284755
NO concen.	9.871E+00	0.003	1.0	0.160	0.692143
CO concen.	6.249E+01	0.019	1.0	1.013	0.322745
Residuals	1.727E+03	0.534	28.0		

*p<0.1; **p<0.05; ***p<0.01

Supplementary Table 23: Mortality by COPD (significant variables)

Variable	Sum Sq	Variance	Df	F value	P-value
Smoking	1.814E+03	0.353	1.0	24.869	0.000016***
Flow-hierarchy	7.041E+02	0.137	1.0	9.651	0.003681**
Residuals	2.626E+03	0.510	36.0		

*p<0.1; **p<0.05; ***p<0.01

Supplementary Table 24: Mortality by all type of strokes (all variables)

Variable	Sum Sq	Variance	Df	F value	P-value
Flow-hierarchy	4.193E+02	0.164	1.0	6.070	0.020154*
Obesity	1.213E+01	0.005	1.0	0.176	0.678410
GDP	2.658E+00	0.001	1.0	0.038	0.845893
Perc. of poverty	1.558E+01	0.006	1.0	0.226	0.638494
Smoking	2.614E+01	0.010	1.0	0.378	0.543392
Alcohol	1.410E+01	0.006	1.0	0.204	0.654843
O_3 concen.	1.135E+01	0.004	1.0	0.164	0.688242
CO concen.	2.276E-01	0.000	1.0	0.003	0.954630
NO concen.	1.135E+02	0.044	1.0	1.643	0.210361
SO concen.	4.595E+00	0.002	1.0	0.067	0.798364
Residuals	1.934E+03	0.757	28.0		

*p<0.1; **p<0.05; ***p<0.01

Supplementary Table 25: Mortality by all type of strokes (significant variables)

Variable	Sum Sq	Variance	Df	F value	P-value
Flow-hierarchy	7.225E+02	0.218	1.0	11.189	0.001933**
Obesity	2.618E+02	0.079	1.0	4.054	0.051591
Residuals	2.325E+03	0.703	36.0		

*p<0.1; **p<0.05; ***p<0.01

Supplementary Table 26: Mortality by ischemic stroke (all variables)

Variable	Sum Sq	Variance	Df	F value	P-value
Flow-hierarchy	6.078E+01	0.089	1.0	3.987	0.055647
Obesity	1.620E+02	0.237	1.0	10.627	0.002925**
GDP	6.234E+00	0.009	1.0	0.409	0.527689
Perc. of poverty	6.982E+00	0.010	1.0	0.458	0.504112
Smoking	2.551E-01	0.000	1.0	0.017	0.898000
Alcohol	1.689E-01	0.000	1.0	0.011	0.916911
O_3 concen.	8.176E+00	0.012	1.0	0.536	0.470045
SO concen.	3.602E-02	0.000	1.0	0.002	0.961572
NO concen.	1.020E+01	0.015	1.0	0.669	0.420173
CO concen.	4.692E-01	0.001	1.0	0.031	0.861988
Residuals	4.268E+02	0.626	28.0		

*p<0.1; **p<0.05; ***p<0.01

Supplementary Table 27: Mortality by ischemic stroke (significant variables)

Variable	Sum Sq	Variance	Df	F value	P-value
Flow-hierarchy	1.133E+02	0.096	1.0	8.552	0.005938**
Obesity	5.933E+02	0.501	1.0	44.788	8.34E-8***
Residuals	4.769E+02	0.403	36.0		

*p<0.1; **p<0.05; ***p<0.01

Supplementary Table 28: Hospitalization by ischemic stroke (significant variables)

Variable	Sum Sq	Variance	Df	F value	P-value
Flow-hierarchy	0.495	0.00580	1	0.6641	0.4205
Obesity	58.025	0.67961	1	77.7732	1.596e-10 ***
Residuals	26.859	0.31458	36		

*p<0.1; **p<0.05; ***p<0.01

Supplementary Table 29: Mortality by transport fatalities (all variables)

Variable	Sum Sq	Variance	Df	F value	P-value
Flow-hierarchy	4.311E+01	0.274	1.0	13.052	0.001025**
Obesity	2.783E-01	0.002	1.0	0.084	0.773453
GDP	5.495E+00	0.035	1.0	1.664	0.206329
Perc. of poverty	1.269E+00	0.008	1.0	0.384	0.539680
Smoking	1.306E+00	0.008	1.0	0.396	0.533845
Alcohol	1.925E-02	0.000	1.0	0.006	0.939621
Residuals	1.057E+02	0.672	32.0		

*p<0.1; **p<0.05; ***p<0.01

Supplementary Table 30: Mortality by transport fatalities (significant variables)

Variable	Sum Sq	Variance	Df	F value	P-value
GDP	6.153E+01	0.281	1.0	20.153	0.000071***
Flow-hierarchy	4.717E+01	0.216	1.0	15.447	0.000369***
Residuals	1.099E+02	0.503	36.0		

*p<0.1; **p<0.05; ***p<0.01

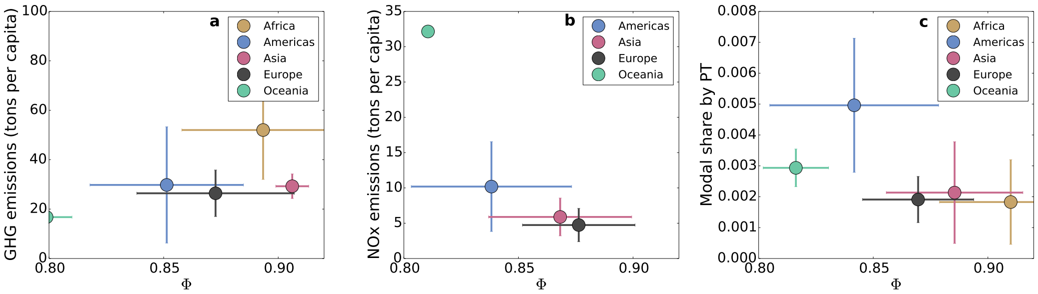

Supplementary Figure 27: Relation between the flow-hierarchy, modal split and pollution averaged per continent. (a) Modal share as a function of the city flow-hierarchy, (b) Greenhouse gas emissions per capita in equivalent metric tons of CO2, (c) NOX emissions per capita.

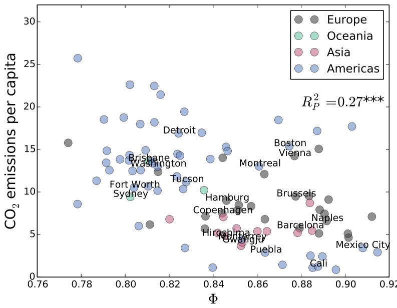

Supplementary Figure 28: Relation between Φ and CO_2 emissions per capita.

Worldwide cities

Here, we show additional results for worldwide cities. In Supplementary Fig. 27, we compare Φ, averaged over continents, to emissions and modal share of transport. The OECD also provides an estimation of CO_2 emissions in a subset of cities. In Supplementary Fig. 28, we show the correlation between the flow-hierarchy and the emissions of CO_2, observing a smaller but still significant correlation. Despite a small group of outliers, the majority of cities are anti-correlated. with a negative correlation. More hierarchical cities have smaller emissions of CO_2.

Supplementary References

[1] The bright side of sitting in traffic: Crowdsourcing road congestion data, http://googlemobile.blogspot.com/2009/08/bright-side-of-sitting-in-traffic.html, August 2009.

[2] Kirmse, Andrew, et al. Extracting patterns from location history. *Proceedings of the 19th ACM SIGSPATIAL International Conference on Advances in Geographic Information Systems. ACM*, (2011).

[3] S2 Geometry | S2Geometry Available at: http://s2geometry.io/ (Accessed: 22nd January 2019)

[4] E. Arcaute, E. Hatna, P. Ferguson, H. Youn, A. Johansson, and M. Batty, M., *Journal of The Royal Society Interface* **12.102**, 20140745 (2015).

[5] C. Cottineau, E. Hatna, E. Arcaute, and M. Batty, *Computers, Environment and Urban Systems* **63**, 80-94 (2017).

[6] Census of Population and Housing - Publications - U.S. Census Bureau Available at: https://www.census.gov/prod/cen2010/doc/sf1.pdf (Accessed: 22nd January 2019)

[7] Cartographic Boundary Shapefiles - Geography - U.S. Census Bureau Available at: https://www.census.gov/geo/maps-data/data/tiger-cart-boundary.html (Accessed: 22nd January 2019)

[8] Functional urban areas by country - OECD Available at: http://www.oecd.org/cfe/regional-policy/functionalurbanareasbycountry.html (Accessed: 22nd January 2019)

[9] The Atlas of Urban Expansion Available at: http://www.atlasofurbanexpansion.org/ (Accessed: 22nd January 2019)

[10] M.C. Gonzalez, C.A. Hidalgo, and A.-L. Barabasi, *Nature* **453**, 779-782 (2008).

[11] D. Brockmann, L. Hufnagel, and T. Geisel, *Nature* **439**, 462-465 (2006).

[12] A. Bazzani, B. Giorgini, S. Rambaldi, R. Gallotti, and L. Giovannini, *Journal of Statistical Mechanics: Theory and Experiment* **2010(05)**, P05001 (2010).

[13] A. Noulas, S. Scellato, R. Lambiotte, M. Pontil, and C. Mascolo, *PloS one*, **7**, e37027 (2012).

[14] J.Tang, F. Liu, Y. Wang, H. Wang, *Physica A: Statistical Mechanics and its Applications.* **438**, 140-153 (2015).

[15] T. Louail et al., *Scientific reports* **4**, 5276 (2014).

[16] V. Volpati and M. Barthelemy, arXiv preprint arXiv:1804.00855 (2018).

[17] L. Bettencourt, J. Lobo, D. Helbing, C. Kuhnert and G. West, *Proc. Natl Acad. Sci. USA* **104**, 7301-7306 (2007).

[18] L. Bettencourt The origins of scaling in cities. *Science* **340**, 1438-1441 (2013).

[19] Alan Wilson. *Entropy in urban and regional modelling*, volume 1. Routledge, 2011.

[20] Sven Erlander and Neil F Stewart. *The gravity model in transportation analysis: theory and extensions*, volume 3. Vsp, 1990.

[21] Maxime Lenormand, Aleix Bassolas, and José J Ramasco. Systematic comparison of trip distribution laws and models. *Journal of Transport Geography*, 51:158–169, 2016.

[22] Filippo Simini, Marta C González, Amos Maritan, and Albert-László Barabási. A universal model for mobility and migration patterns. *Nature*, 484(7392):96, 2012.

[23] Xiao-Yong Yan, Chen Zhao, Ying Fan, Zengru Di, and Wen-Xu Wang. Universal predictability of mobility patterns in cities. *Journal of The Royal Society Interface*, 11(100):20140834, 2014.

[24] Bettencourt, L. M. The origins of scaling in cities. *Science* **340**, 1438–1441 (2013).

[25] Louail, T., Lenormand, M., Murillo Arias, J. & Ramasco, J. J. Crowdsourcing the Robin Hood effect in cities. *Applied Network Science* **2**, 11 (2017).

[26] American FactFinder - Census Bureau Available at: https://factfinder.census.gov/faces/nav/jsf/pages/index.xhtml (Accessed: 22nd January 2019)

[27] Behavioral Risk Factor Surveillance System (BRFSS) Prevalence Data (2011 to present) Available at: https://chronicdata.cdc.gov/Behavioral-Risk-Factors/Behavioral-Risk-Factor-Surveillance-System-BRFSS-P/dttw-5yxu (Accessed: 22nd January 2019)

[28] 2014 National Emissions Inventory (NEI) Data. EPA (2018). Available at: https://www.epa.gov/air-emissions-inventories/2014-national-emissions-inventory-nei-data. (Accessed: 22nd January 2019)

[29] OMB Bulletin 15-01, Revised Delineations of Metropolitan Statistical Areas, Micropolitan Statistical Areas, and Combined Statistical Areas, and Guidance on Uses of the Delineations of These Areas Available at: https://web.archive.org/web/20161110102915/https://www.whitehouse.gov/sites/default/files/omb/bulletins/2015/1 01.pdf (Accessed: 22nd January 2019)

[30] Interactive Atlas of Heart Disease and Stroke - CDC Available at: https://nccd.cdc.gov/dhdspatlas/reports.aspx (Accessed: 22nd January 2019)

[31] Transportation and Health Tool Available at: https://www.transportation.gov/transportation-health-tool/indicators (Accessed: 22nd January 2019)

[32] Institute for Health Metrics and Evaluation Available at: http://www.healthdata.org/ (Accessed: 22nd January 2019)

[33] CDC Tracking Network Available at: https://ephtracking.cdc.gov/DataExplorer/ (Accessed: 22nd January 2019)

[34] Homeland Infrastructure Foundation-Level Data (HIFLD) Available at: https://hifld-geoplatform.opendata.arcgis.com/datasets/hospitals (Accessed: 22nd January 2019)

[35] Global BRTData Available at: https://brtdata.org/ (Accessed: 22nd January 2019)

[36] CDP Disclosure Insight Action Available at: https://www.cdp.net/es (Accessed: 22nd January 2019)

[37] Measuring Urban (OECD) Available at: https://measuringurban.oecd.org/ (Accessed: 22nd January 2019)

[38] Emission Database for Global Atmospheric Research (EDGAR) Available at: http://edgar.jrc.ec.europa.eu (Accessed: 22nd January 2019)

[39] C. K. Gately, L. R. Hutyra and I. S. Wing, *Proc. Natl. Acad. Sci. USA* **112**, 4999-5004 (2015).

[40] R. B. Cleveland, W. S. Cleveland, J. E. McRae, and I. Terpenning, *Journal of Official Statistics*, 6:3-33 (1990).

[41] W. S. Cleveland and S. J. Devlin, *Journal of the American Statistical Association*, 83:596610 (1988).

[42] W. M. Shyu, E. Grosse, and W. S. Cleveland, In *Statistical models in S.* Routledge Chapman & Hall, New York 309-376 (2017).

[43] C.R. Loader, *Springer* (1999).

[44] W.G. Jacoby, *Electoral Studies* **19.4**, 577-613 (2000).

[45] R. Ewing, R. Pendall, & Chen, D. Measuring sprawl and its transportation impacts. *TRR: JTRB* **183**, 175-183 (2003).

[46] P. Newman and J. R. Kenworthy, *Island Press*, Washington DC (2015) .

[47] C. Kennedy et al., Greenhouse gas emissions from global cities. 7297-7302 (2009).

[48] R. Ewing, R. A. Schieber and C. V. Zegeer Urban sprawl as a risk factor in motor vehicle occupant and pedestrian fatalities. *American journal of public health* **93.9**, 1541-1545(2003).

[49] E.A. LaScala, D. Gerber, and P.J. Gruenewald, Demographic and environmental correlates of pedestrian injury collisions: a spatial analysis. *Accident Analysis* & *Prevention* **32.5**, 651-658(2000).

[50] R. Ewing, and S. Hamidi Compactness versus sprawl: A review of recent evidence from the United States. *Journal of Planning Literature* **30.4**, 413-432 (2015).

[51] R. Ewing and S. Hamidi, Measuring Sprawl 2014. Available at: www.smartgrowthamerica.org/measuring-sprawl. (Accessed: 22nd January 2019)

[52] J. Um et al., *Proc. Natl. Acad. Sci. U.S.A.* **106.34**, 14236-14240 (2009).
